# Supplementary material for: High-content screening identifies a small molecule that restores AP-4-dependent protein trafficking in neuronal models of AP-4-associated hereditary spastic paraplegia
Source: Nat Commun. 2024 Jan 17;15:584. doi: 10.1038/s41467-023-44264-1 (PMC10794252; doi:10.1038/s41467-023-44264-1)
Supplement: Supplementary file 1 — Supplementary Information [file 41467_2023_44264_MOESM1_ESM.pdf]

Supplementary Figure 1.

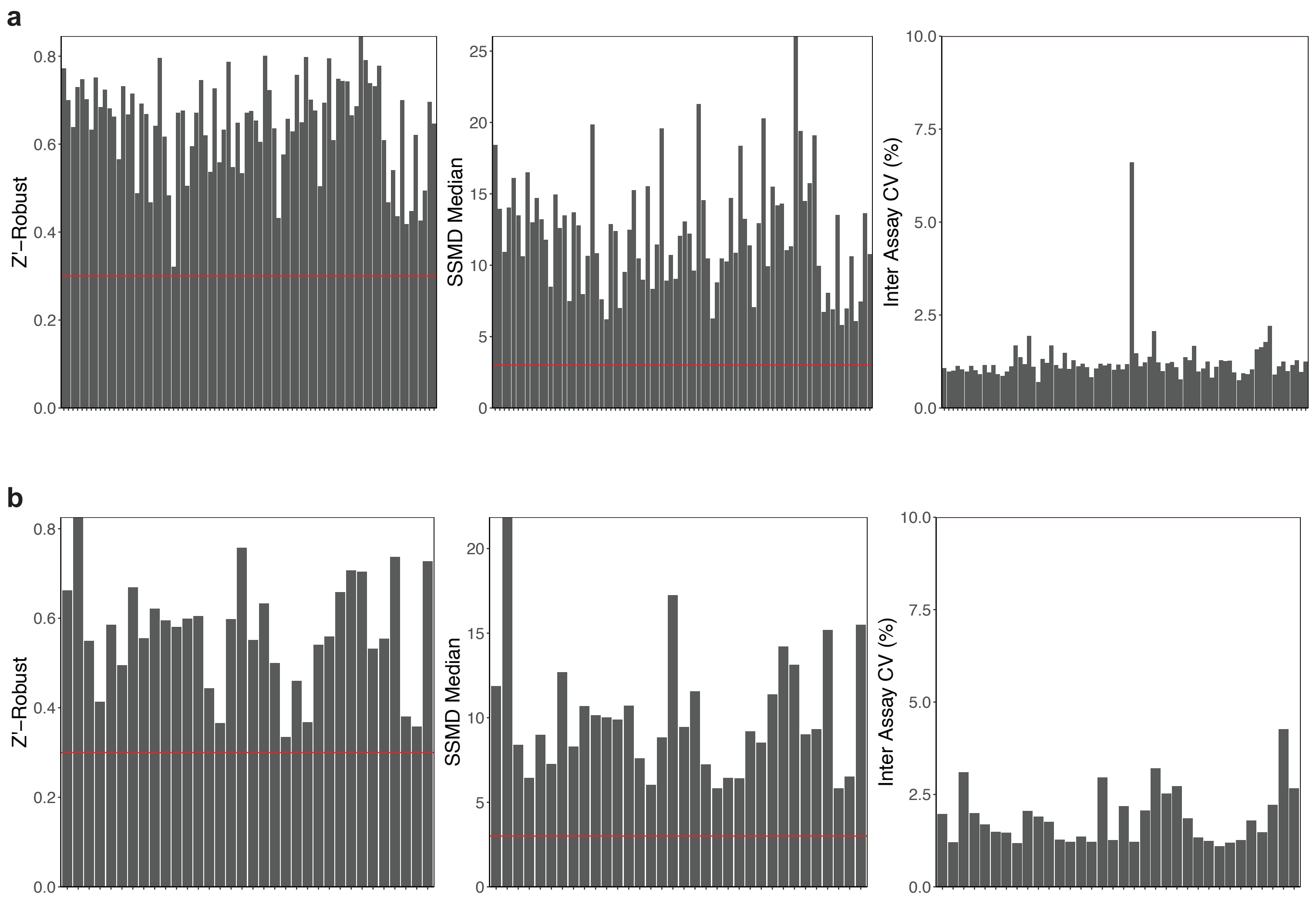

Supplementary Figure 1. Quality metrics of the ATG9A translocation assay in the primary screen and counter-screen.

Assay performance was monitored in the (a) primary screen and (b) counter-screen using criteria proposed by Zhang *et al.* and included a Z' robust ≥ 0.3, a strictly standardized median difference (SSMD) ≥ 3 and an inter-assay coefficient of variation $\leq 10\%$. All metrics were calculated with respect to the positive and negative controls of the same assay plate to avoid bias by inter-plate variability. Predefined thresholds (red lines) were met by all assay plates.

Supplementary Figure 2.

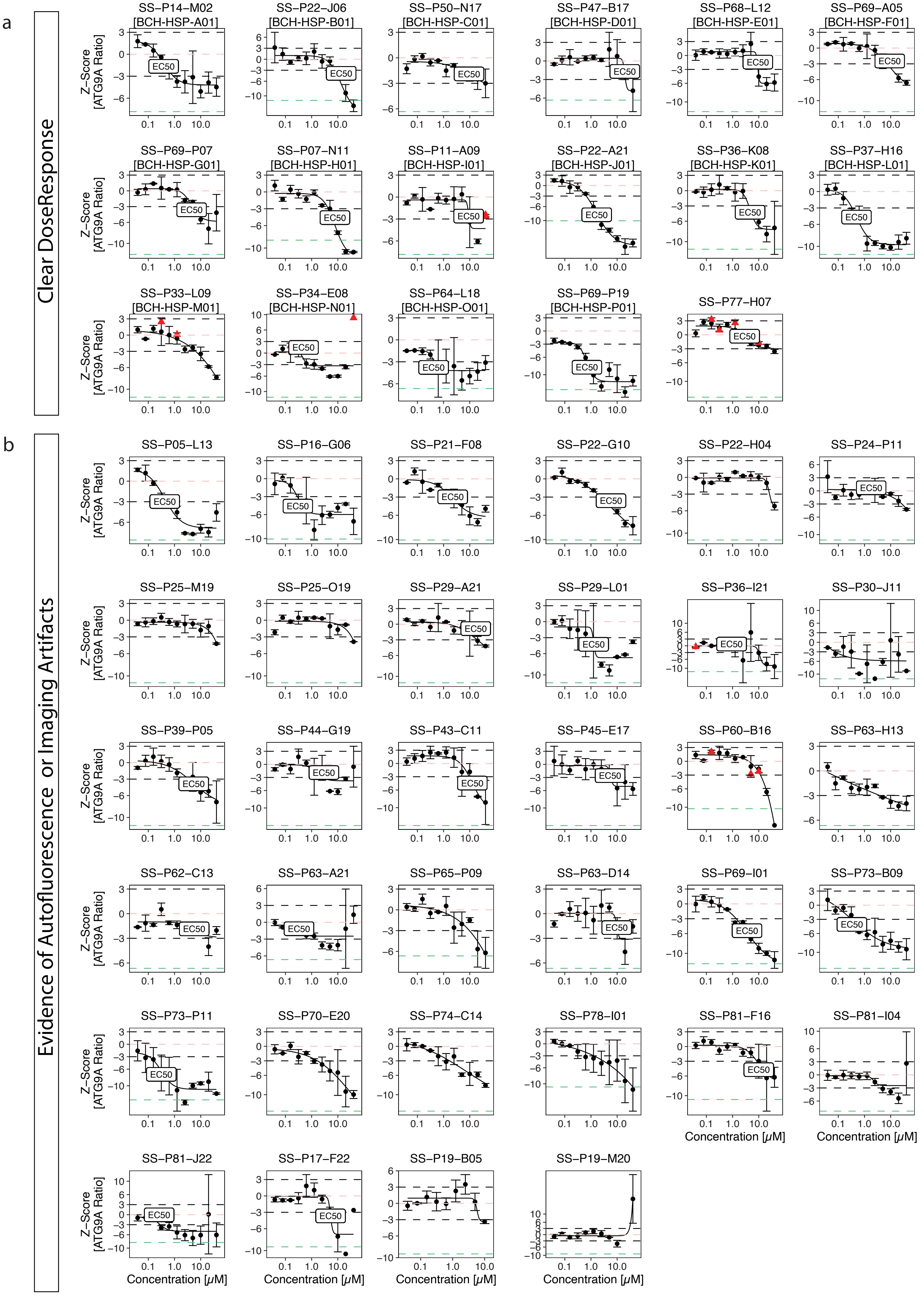

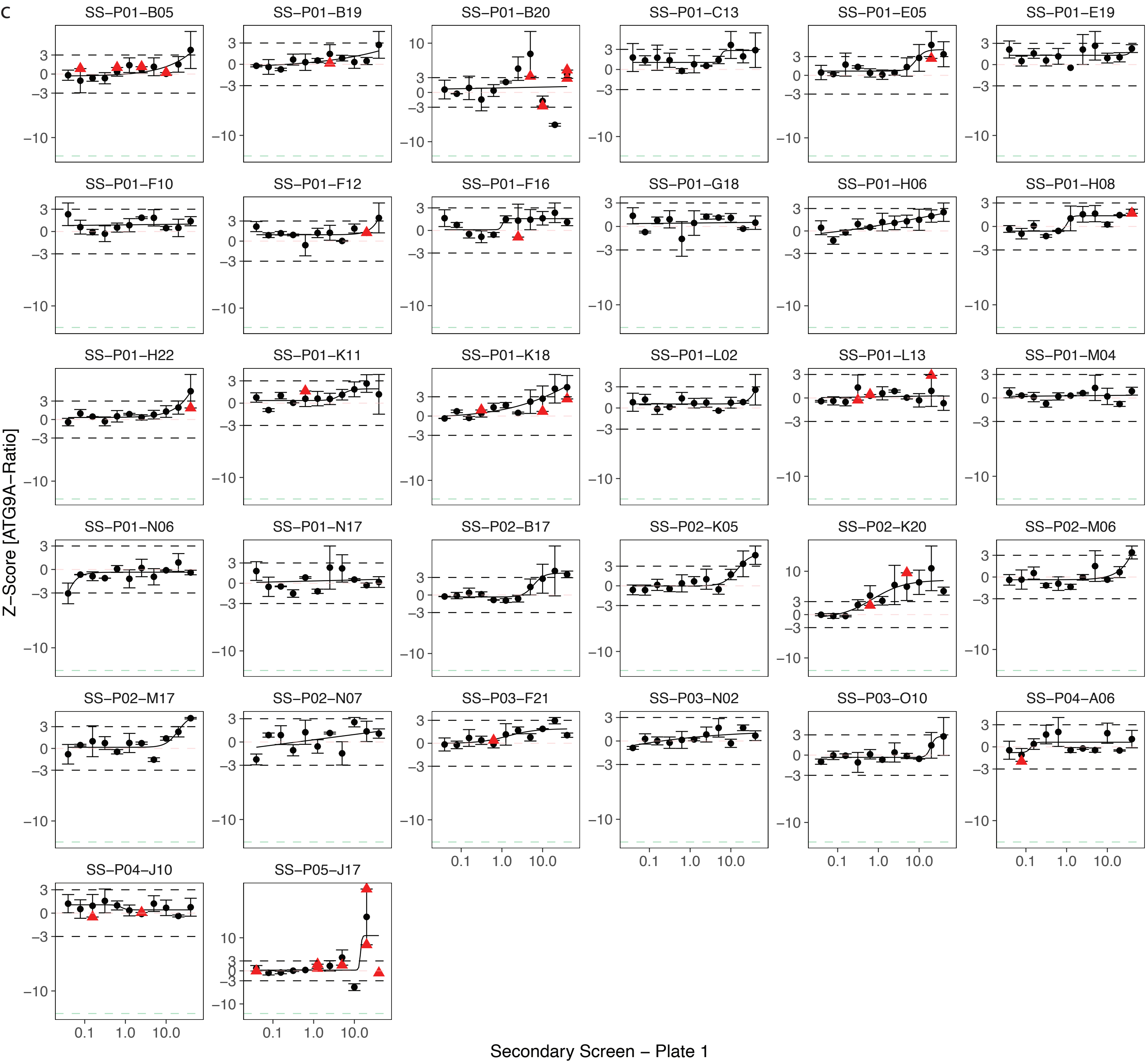
C
SS–P01–B05
SS–P01–B19
SS–P01–B20
SS–P01–C13
SS–P01–E05
SS–P01–E19
SS–P01–F10
SS–P01–F12
SS–P01–F16
SS–P01–G18
SS–P01–H06
SS–P01–H08
SS–P01–H22
SS–P01–K11
SS–P01–K18
SS–P01–L02
SS–P01–L13
SS–P01–M04
SS–P01–N06
SS–P01–N17
SS–P02–B17
SS–P02–K05
SS–P02–K20
SS–P02–M06
SS–P02–M17
SS–P02–N07
SS–P03–F21
SS–P03–N02
SS–P03–O10
SS–P04–A06
SS–P04–J10
SS–P05–J17
Z–Score [ATG9A–Ratio]
Secondary Screen – Plate 1

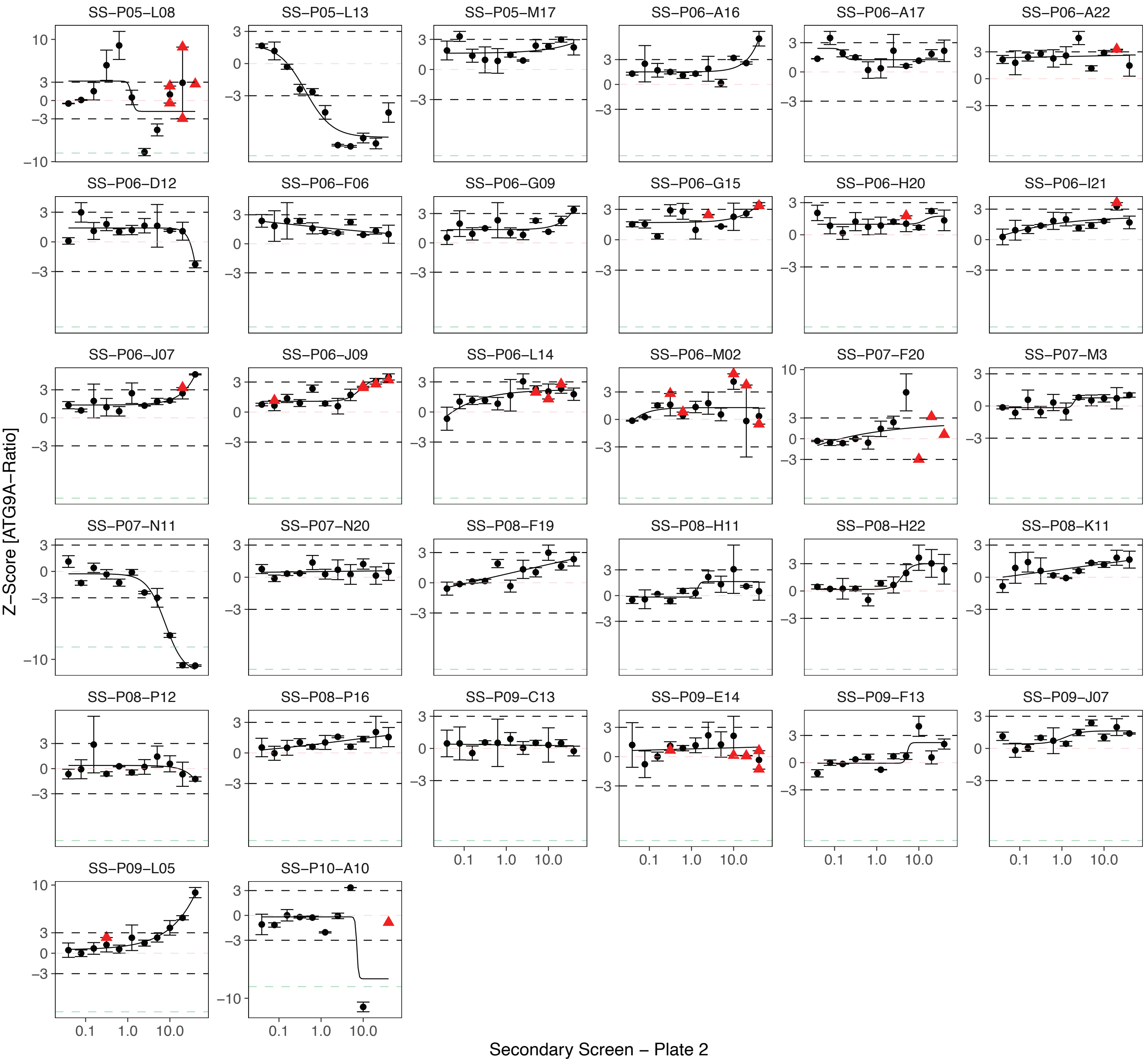

SS–P05–L08
SS–P05–L13
SS–P05–M17
SS–P06–A16
SS–P06–A17
SS–P06–A22
SS–P06–D12
SS–P06–F06
SS–P06–G09
SS–P06–G15
SS–P06–H20
SS–P06–I21
SS–P06–J07
SS–P06–J09
SS–P06–L14
SS–P06–M02
SS–P07–F20
SS–P07–M3
SS–P07–N11
SS–P07–N20
SS–P08–F19
SS–P08–H11
SS–P08–H22
SS–P08–K11
SS–P08–P12
SS–P08–P16
SS–P09–C13
SS–P09–E14
SS–P09–F13
SS–P09–J07
SS–P09–L05
SS–P10–A10
Z–Score [ATG9A–Ratio]
0.1
1.0
10.0
Secondary Screen – Plate 2

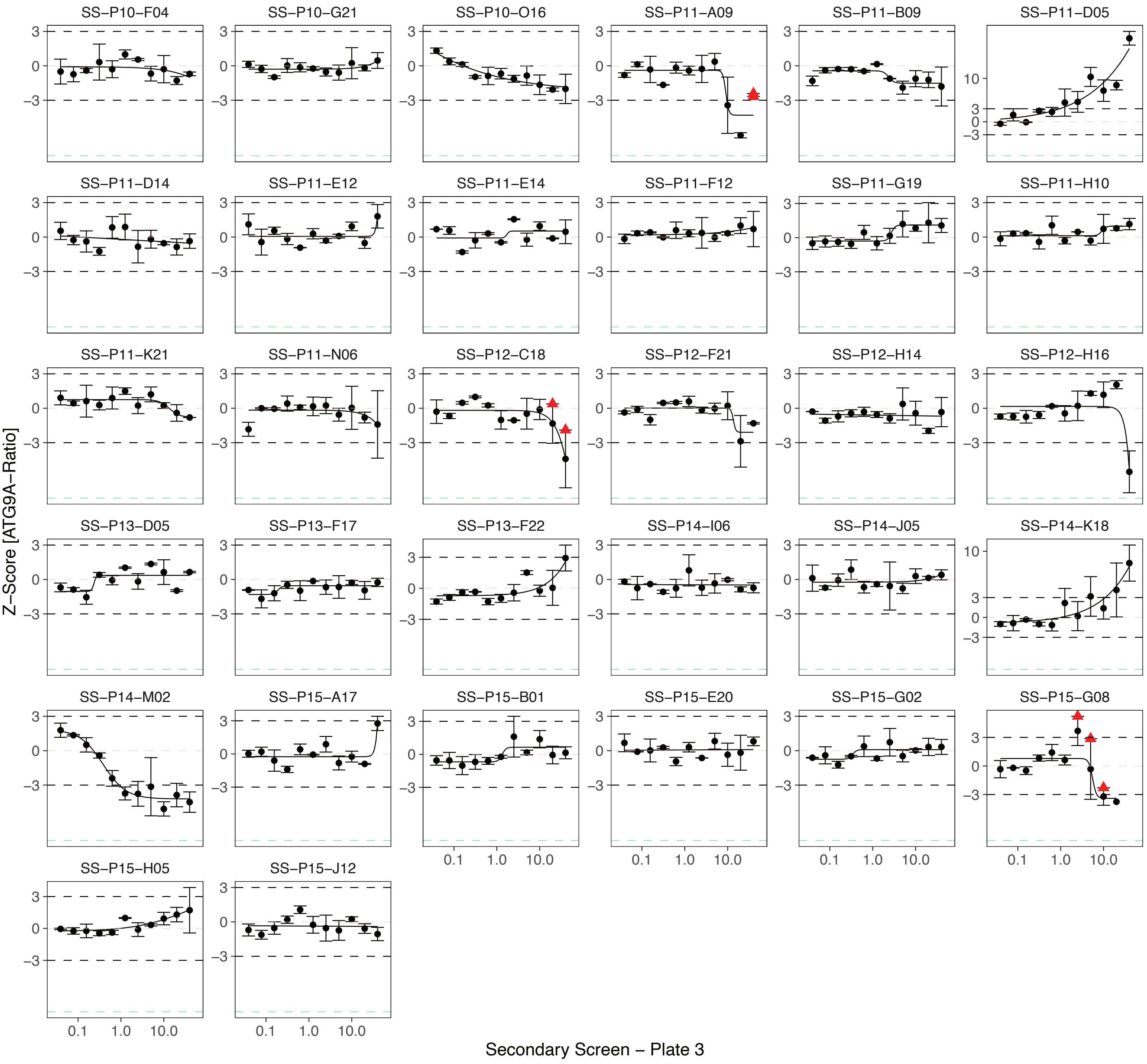
SS–P10–F04
SS–P10–G21
SS–P10–O16
SS–P11–A09
SS–P11–B09
SS–P11–D05
SS–P11–D14
SS–P11–E12
SS–P11–E14
SS–P11–F12
SS–P11–G19
SS–P11–H10
SS–P11–K21
SS–P11–N06
SS–P12–C18
SS–P12–F21
SS–P12–H14
SS–P12–H16
SS–P13–D05
SS–P13–F17
SS–P13–F22
SS–P14–I06
SS–P14–J05
SS–P14–K18
SS–P14–M02
SS–P15–A17
SS–P15–B01
SS–P15–E20
SS–P15–G02
SS–P15–G08
SS–P15–H05
SS–P15–J12
Z–Score [ATG9A–Ratio]
3
0
−3
10
0.1
1.0
10.0
Secondary Screen – Plate 3

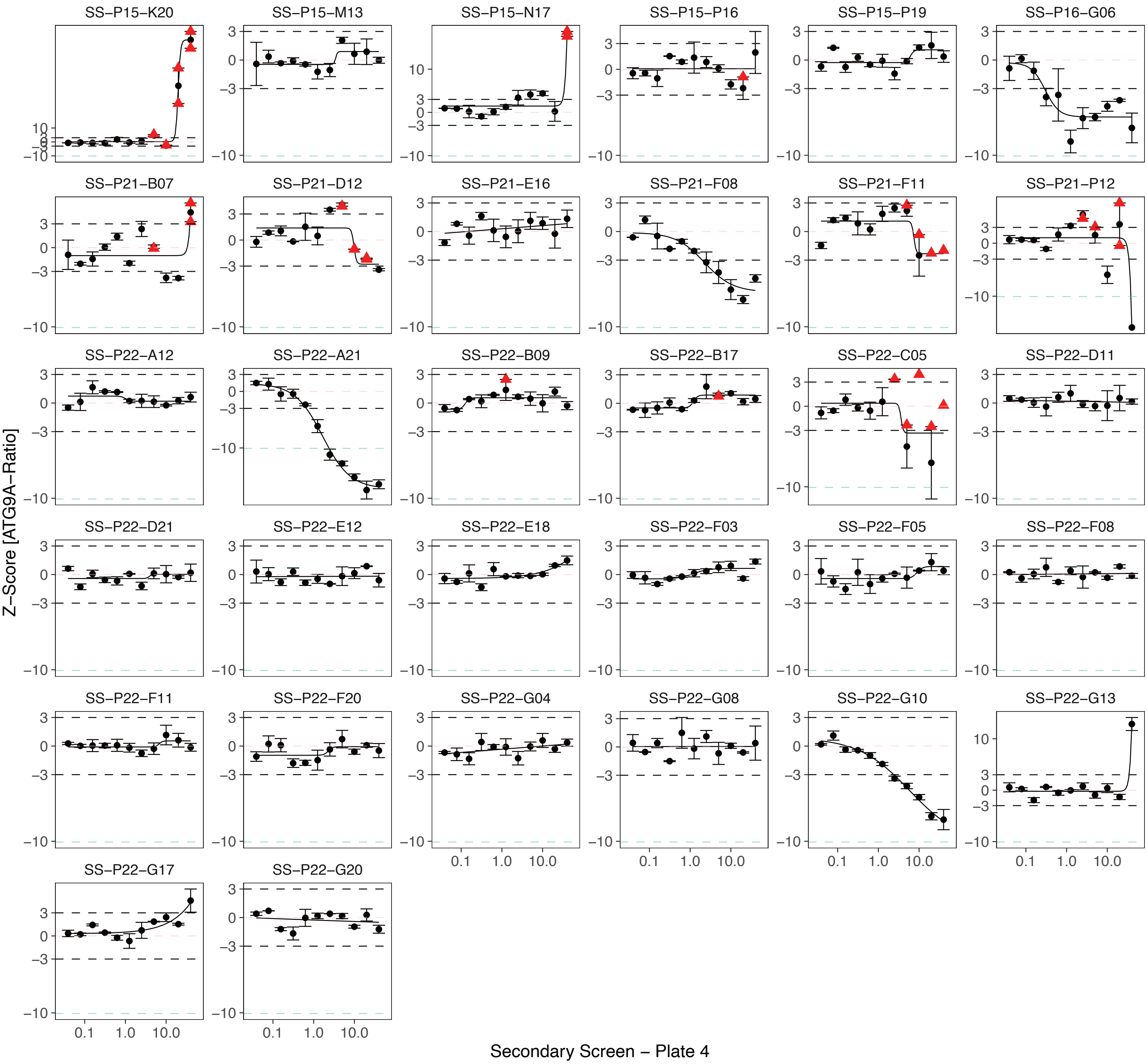

SS–P15–K20
SS–P15–M13
SS–P15–N17
SS–P15–P16
SS–P15–P19
SS–P16–G06
SS–P21–B07
SS–P21–D12
SS–P21–E16
SS–P21–F08
SS–P21–F11
SS–P21–P12
SS–P22–A12
SS–P22–A21
SS–P22–B09
SS–P22–B17
SS–P22–C05
SS–P22–D11
SS–P22–D21
SS–P22–E12
SS–P22–E18
SS–P22–F03
SS–P22–F05
SS–P22–F08
SS–P22–F11
SS–P22–F20
SS–P22–G04
SS–P22–G08
SS–P22–G10
SS–P22–G13
SS–P22–G17
SS–P22–G20
Z–Score [ATG9A–Ratio]
0.1
1.0
10.0
Secondary Screen – Plate 4

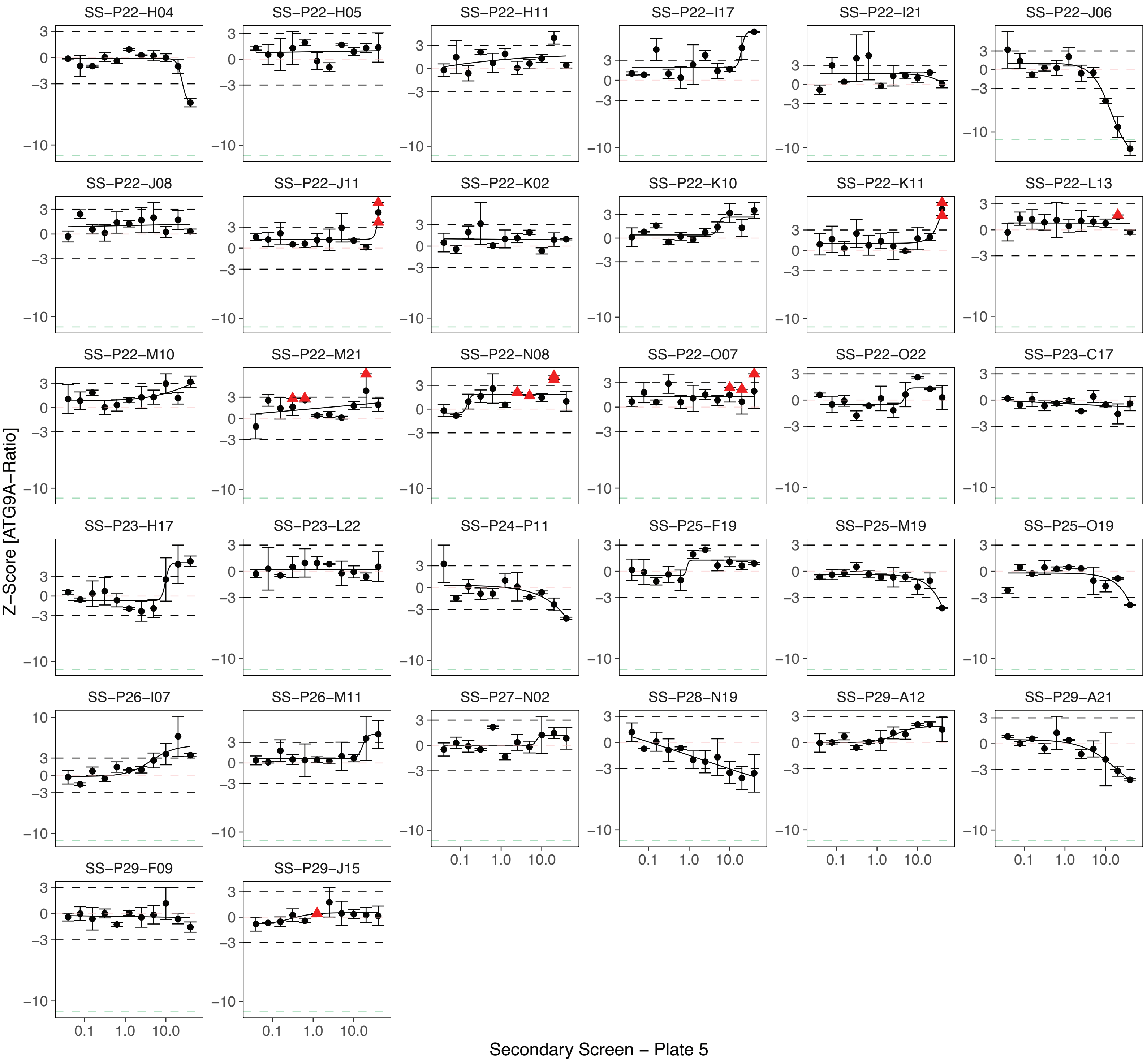
SS−P22−H04
SS−P22−H05
SS−P22−H11
SS−P22−I17
SS−P22−I21
SS−P22−J06
SS−P22−J08
SS−P22−J11
SS−P22−K02
SS−P22−K10
SS−P22−K11
SS−P22−L13
SS−P22−M10
SS−P22−M21
SS−P22−N08
SS−P22−O07
SS−P22−O22
SS−P23−C17
SS−P23−H17
SS−P23−L22
SS−P24−P11
SS−P25−F19
SS−P25−M19
SS−P25−O19
SS−P26−I07
SS−P26−M11
SS−P27−N02
SS−P28−N19
SS−P29−A12
SS−P29−A21
SS−P29−F09
SS−P29−J15
Z−Score [ATG9A−Ratio]
0.1
1.0
10.0
Secondary Screen − Plate 5

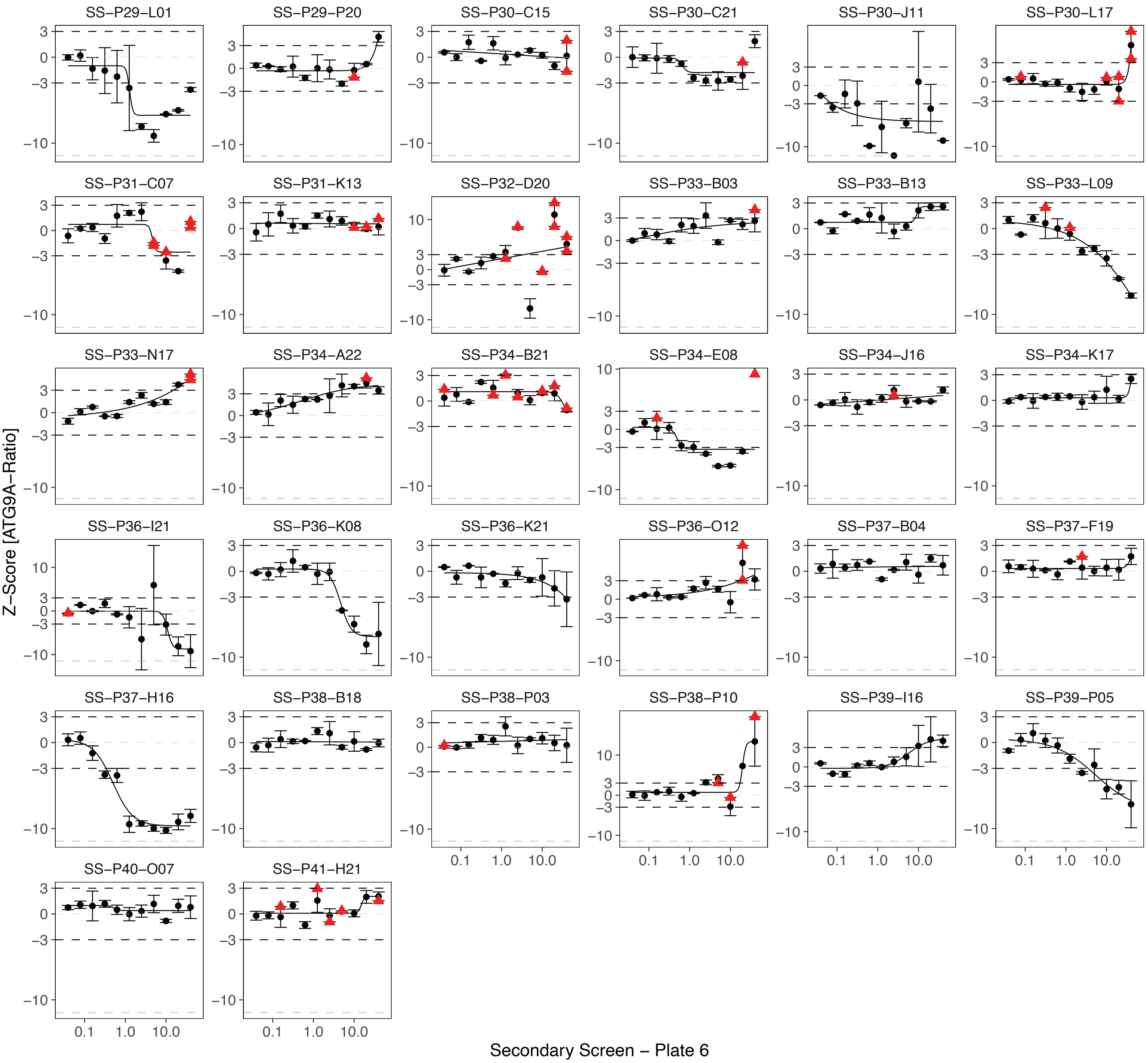
SS−P29−L01
SS−P29−P20
SS−P30−C15
SS−P30−C21
SS−P30−J11
SS−P30−L17
SS−P31−C07
SS−P31−K13
SS−P32−D20
SS−P33−B03
SS−P33−B13
SS−P33−L09
SS−P33−N17
SS−P34−A22
SS−P34−B21
SS−P34−E08
SS−P34−J16
SS−P34−K17
SS−P36−I21
SS−P36−K08
SS−P36−K21
SS−P36−O12
SS−P37−B04
SS−P37−F19
SS−P37−H16
SS−P38−B18
SS−P38−P03
SS−P38−P10
SS−P39−I16
SS−P39−P05
SS−P40−O07
SS−P41−H21
Z−Score [ATG9A−Ratio]
0.1
1.0
10.0
Secondary Screen − Plate 6

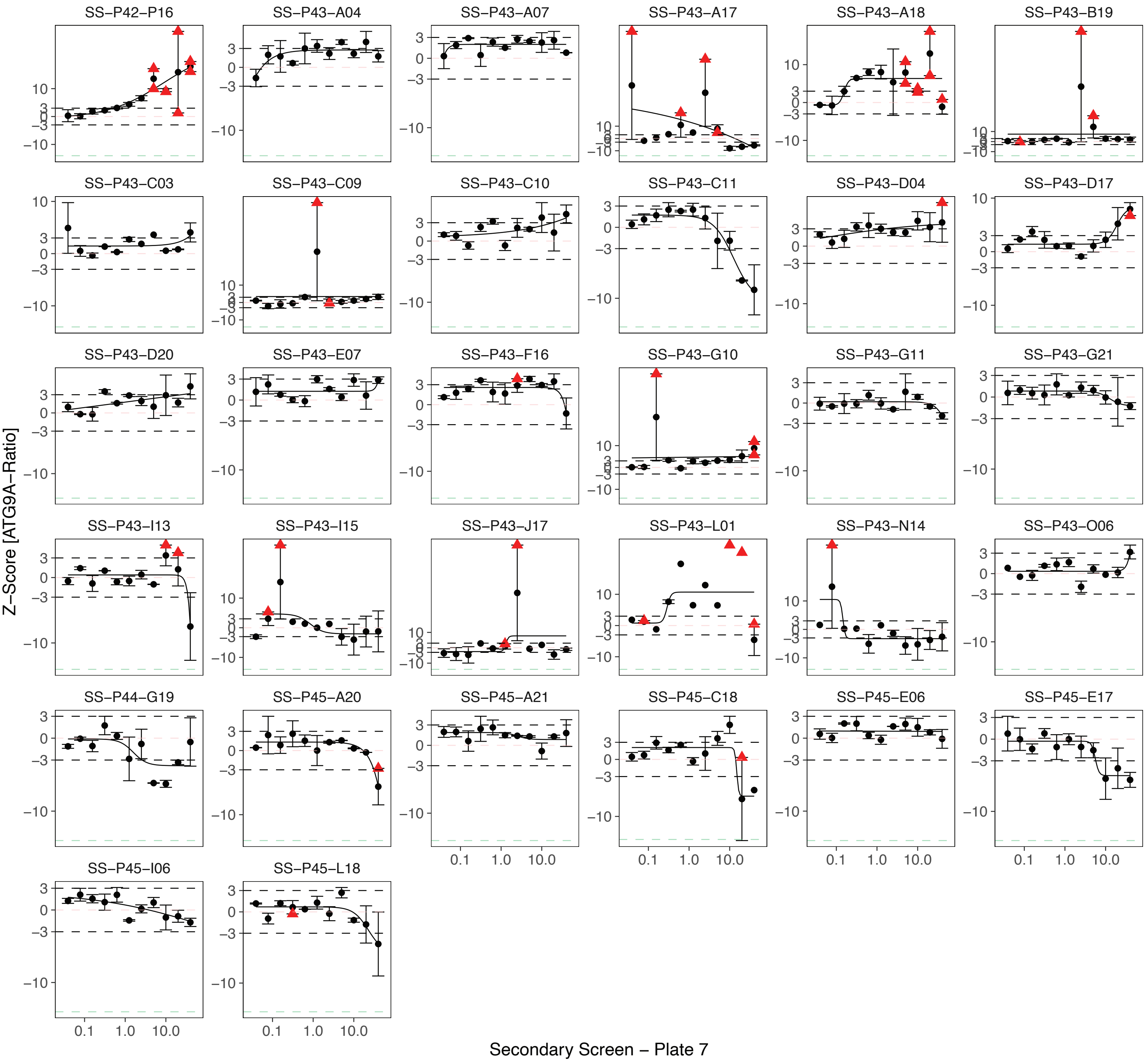
SS–P42–P16
SS–P43–A04
SS–P43–A07
SS–P43–A17
SS–P43–A18
SS–P43–B19
SS–P43–C03
SS–P43–C09
SS–P43–C10
SS–P43–C11
SS–P43–D04
SS–P43–D17
SS–P43–D20
SS–P43–E07
SS–P43–F16
SS–P43–G10
SS–P43–G11
SS–P43–G21
SS–P43–I13
SS–P43–I15
SS–P43–J17
SS–P43–L01
SS–P43–N14
SS–P43–O06
SS–P44–G19
SS–P45–A20
SS–P45–A21
SS–P45–C18
SS–P45–E06
SS–P45–E17
SS–P45–I06
SS–P45–L18
Z–Score [ATG9A–Ratio]
0.1
1.0
10.0
Secondary Screen – Plate 7

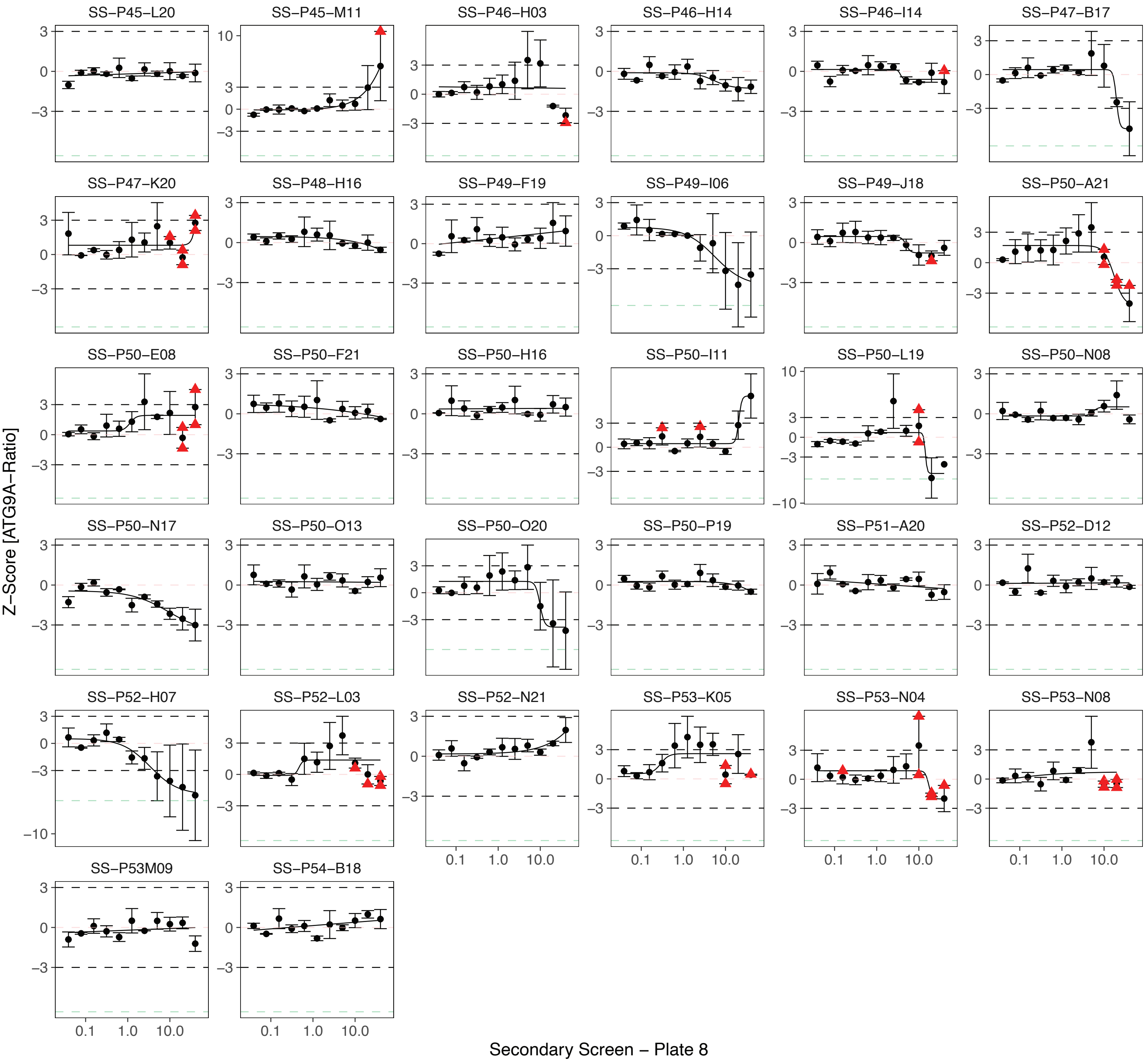
Z−Score [ATG9A−Ratio]
SS−P45−L20
SS−P45−M11
SS−P46−H03
SS−P46−H14
SS−P46−I14
SS−P47−B17
SS−P47−K20
SS−P48−H16
SS−P49−F19
SS−P49−I06
SS−P49−J18
SS−P50−A21
SS−P50−E08
SS−P50−F21
SS−P50−H16
SS−P50−I11
SS−P50−L19
SS−P50−N08
SS−P50−N17
SS−P50−O13
SS−P50−O20
SS−P50−P19
SS−P51−A20
SS−P52−D12
SS−P52−H07
SS−P52−L03
SS−P52−N21
SS−P53−K05
SS−P53−N04
SS−P53−N08
SS−P53M09
SS−P54−B18
0.1
1.0
10.0
Secondary Screen − Plate 8

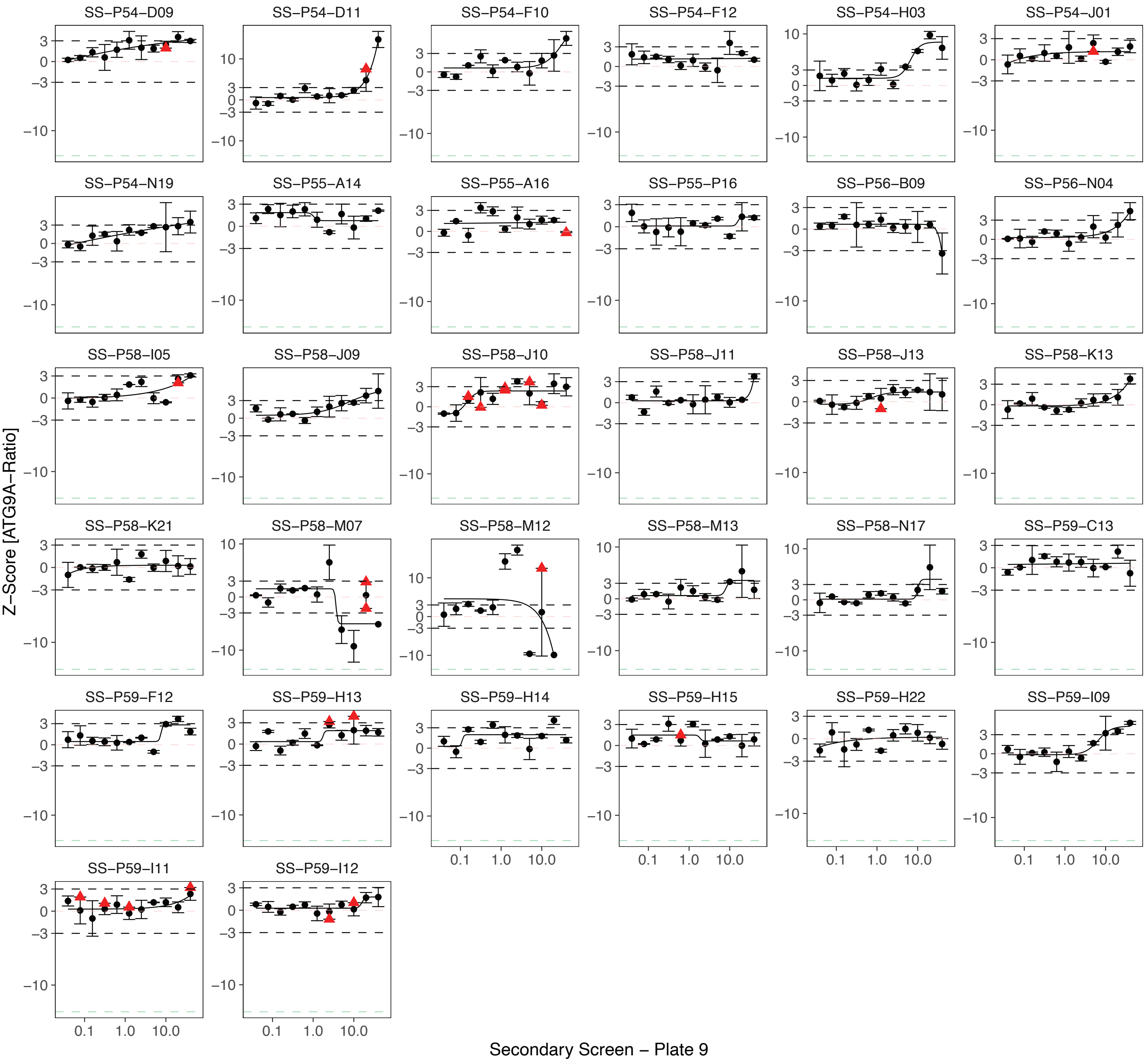

SS–P54–D09
SS–P54–D11
SS–P54–F10
SS–P54–F12
SS–P54–H03
SS–P54–J01
SS–P54–N19
SS–P55–A14
SS–P55–A16
SS–P55–P16
SS–P56–B09
SS–P56–N04
SS–P58–I05
SS–P58–J09
SS–P58–J10
SS–P58–J11
SS–P58–J13
SS–P58–K13
SS–P58–K21
SS–P58–M07
SS–P58–M12
SS–P58–M13
SS–P58–N17
SS–P59–C13
SS–P59–F12
SS–P59–H13
SS–P59–H14
SS–P59–H15
SS–P59–H22
SS–P59–I09
SS–P59–I11
SS–P59–I12
Z–Score [ATG9A–Ratio]
3
0
−3
−10
0.1
1.0
10.0
Secondary Screen – Plate 9

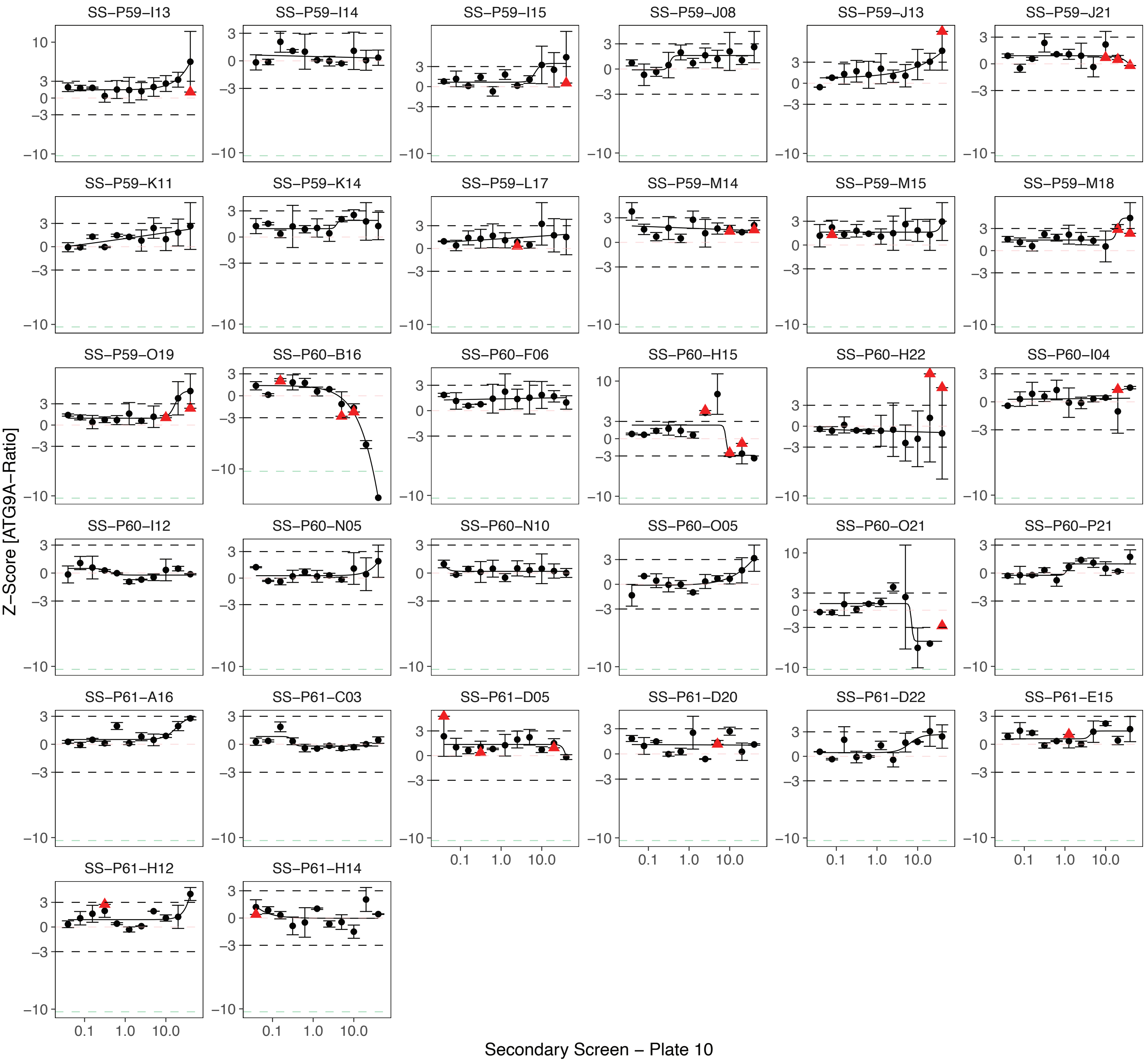

SS−P59−I13
SS−P59−I14
SS−P59−I15
SS−P59−J08
SS−P59−J13
SS−P59−J21
SS−P59−K11
SS−P59−K14
SS−P59−L17
SS−P59−M14
SS−P59−M15
SS−P59−M18
SS−P59−O19
SS−P60−B16
SS−P60−F06
SS−P60−H15
SS−P60−H22
SS−P60−I04
SS−P60−I12
SS−P60−N05
SS−P60−N10
SS−P60−O05
SS−P60−O21
SS−P60−P21
SS−P61−A16
SS−P61−C03
SS−P61−D05
SS−P61−D20
SS−P61−D22
SS−P61−E15
SS−P61−H12
SS−P61−H14
Z−Score [ATG9A−Ratio]
0.1
1.0
10.0
Secondary Screen − Plate 10

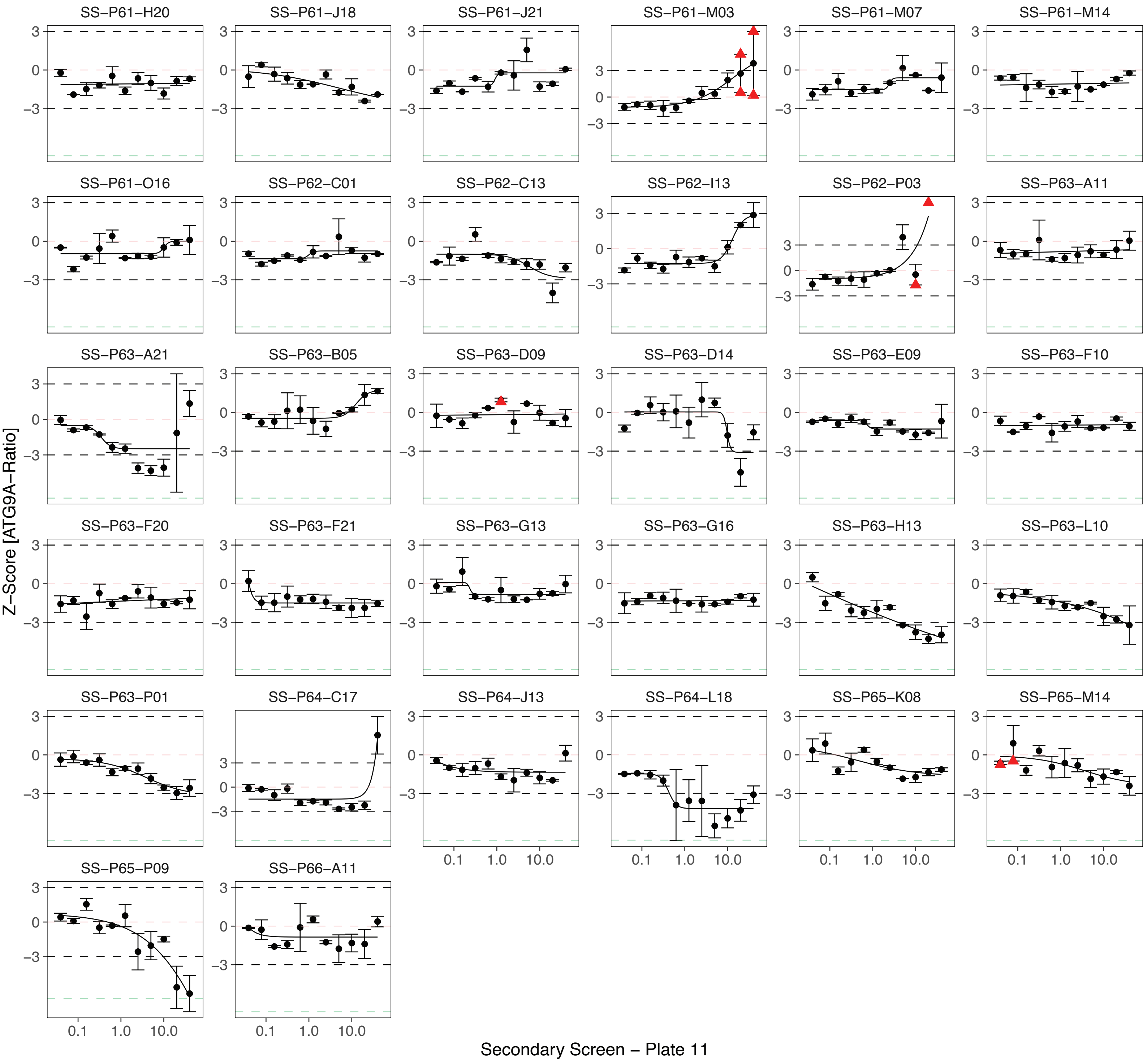
SS–P61–H20
SS–P61–J18
SS–P61–J21
SS–P61–M03
SS–P61–M07
SS–P61–M14
SS–P61–O16
SS–P62–C01
SS–P62–C13
SS–P62–I13
SS–P62–P03
SS–P63–A11
SS–P63–A21
SS–P63–B05
SS–P63–D09
SS–P63–D14
SS–P63–E09
SS–P63–F10
SS–P63–F20
SS–P63–F21
SS–P63–G13
SS–P63–G16
SS–P63–H13
SS–P63–L10
SS–P63–P01
SS–P64–C17
SS–P64–J13
SS–P64–L18
SS–P65–K08
SS–P65–M14
SS–P65–P09
SS–P66–A11
Z–Score [ATG9A–Ratio]
3
0
–3
0.1
1.0
10.0
Secondary Screen – Plate 11

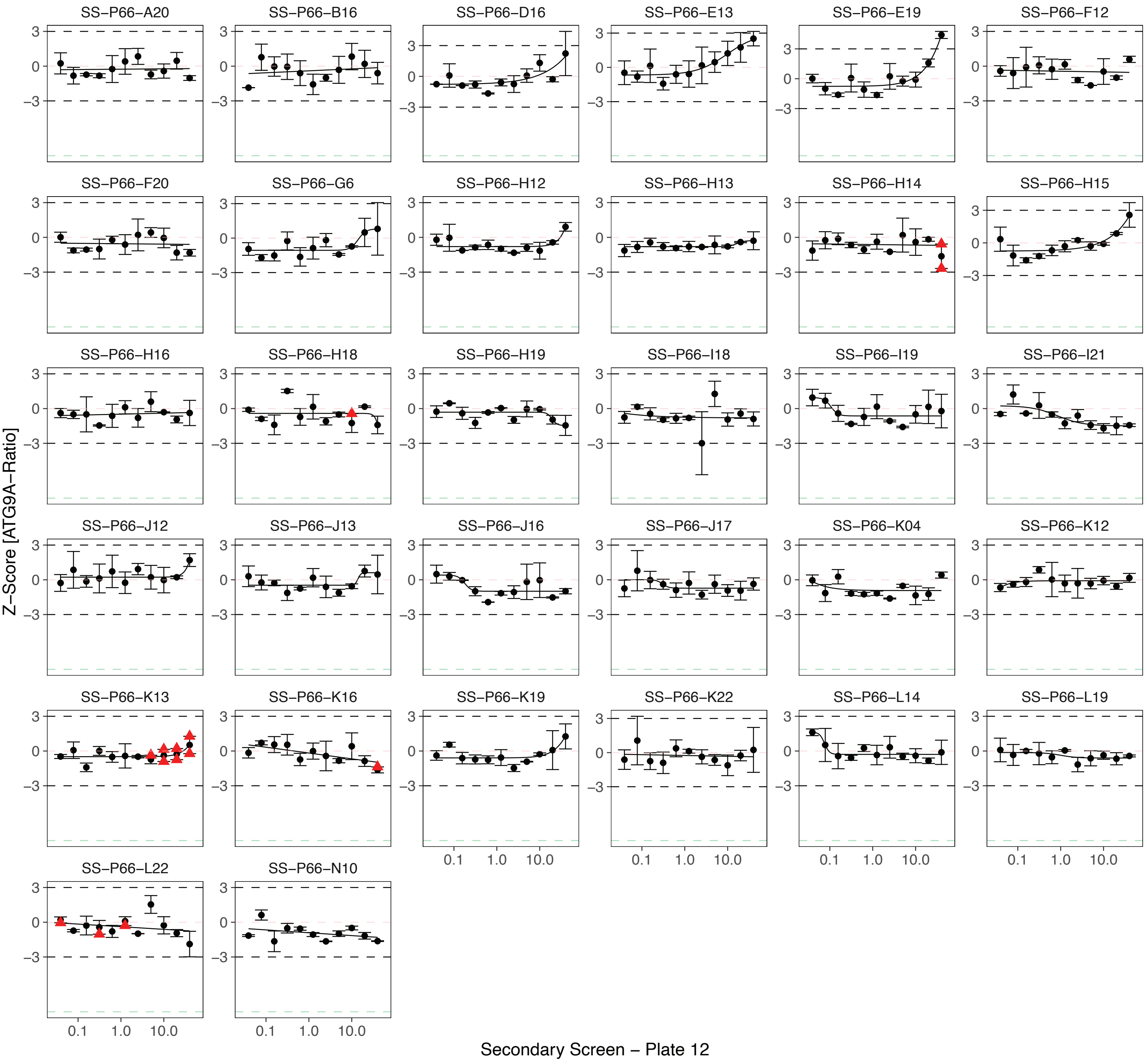
SS−P66−A20
SS−P66−B16
SS−P66−D16
SS−P66−E13
SS−P66−E19
SS−P66−F12
SS−P66−F20
SS−P66−G6
SS−P66−H12
SS−P66−H13
SS−P66−H14
SS−P66−H15
SS−P66−H16
SS−P66−H18
SS−P66−H19
SS−P66−I18
SS−P66−I19
SS−P66−I21
SS−P66−J12
SS−P66−J13
SS−P66−J16
SS−P66−J17
SS−P66−K04
SS−P66−K12
SS−P66−K13
SS−P66−K16
SS−P66−K19
SS−P66−K22
SS−P66−L14
SS−P66−L19
SS−P66−L22
SS−P66−N10
3
0
−3
0.1
1.0
10.0
Z−Score [ATG9A−Ratio]
Secondary Screen − Plate 12

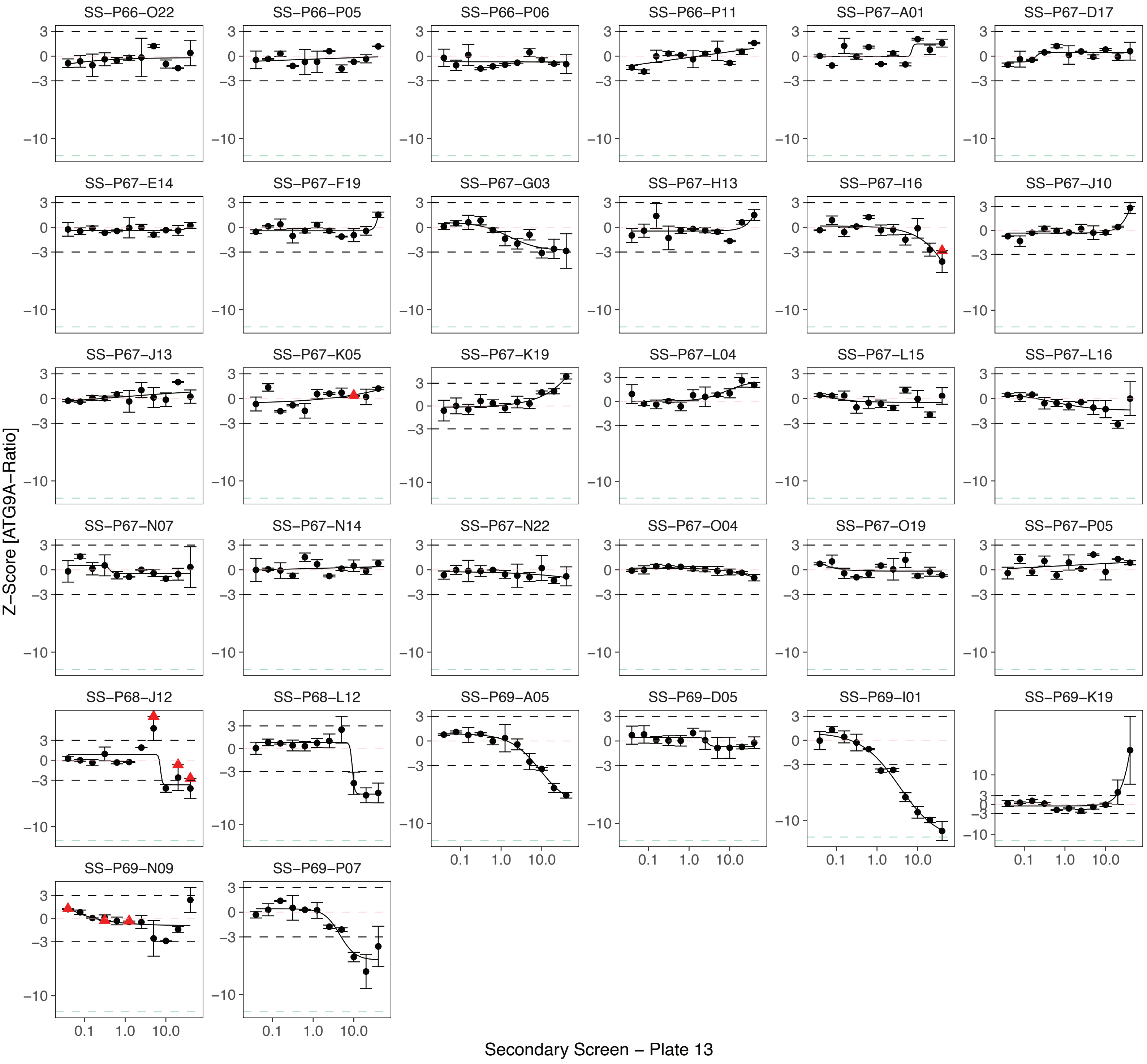

SS−P66−O22
SS−P66−P05
SS−P66−P06
SS−P66−P11
SS−P67−A01
SS−P67−D17
SS−P67−E14
SS−P67−F19
SS−P67−G03
SS−P67−H13
SS−P67−I16
SS−P67−J10
SS−P67−J13
SS−P67−K05
SS−P67−K19
SS−P67−L04
SS−P67−L15
SS−P67−L16
SS−P67−N07
SS−P67−N14
SS−P67−N22
SS−P67−O04
SS−P67−O19
SS−P67−P05
SS−P68−J12
SS−P68−L12
SS−P69−A05
SS−P69−D05
SS−P69−I01
SS−P69−K19
SS−P69−N09
SS−P69−P07
Z−Score [ATG9A−Ratio]
3
0
−3
−10
10
0.1
1.0
10.0
Secondary Screen − Plate 13

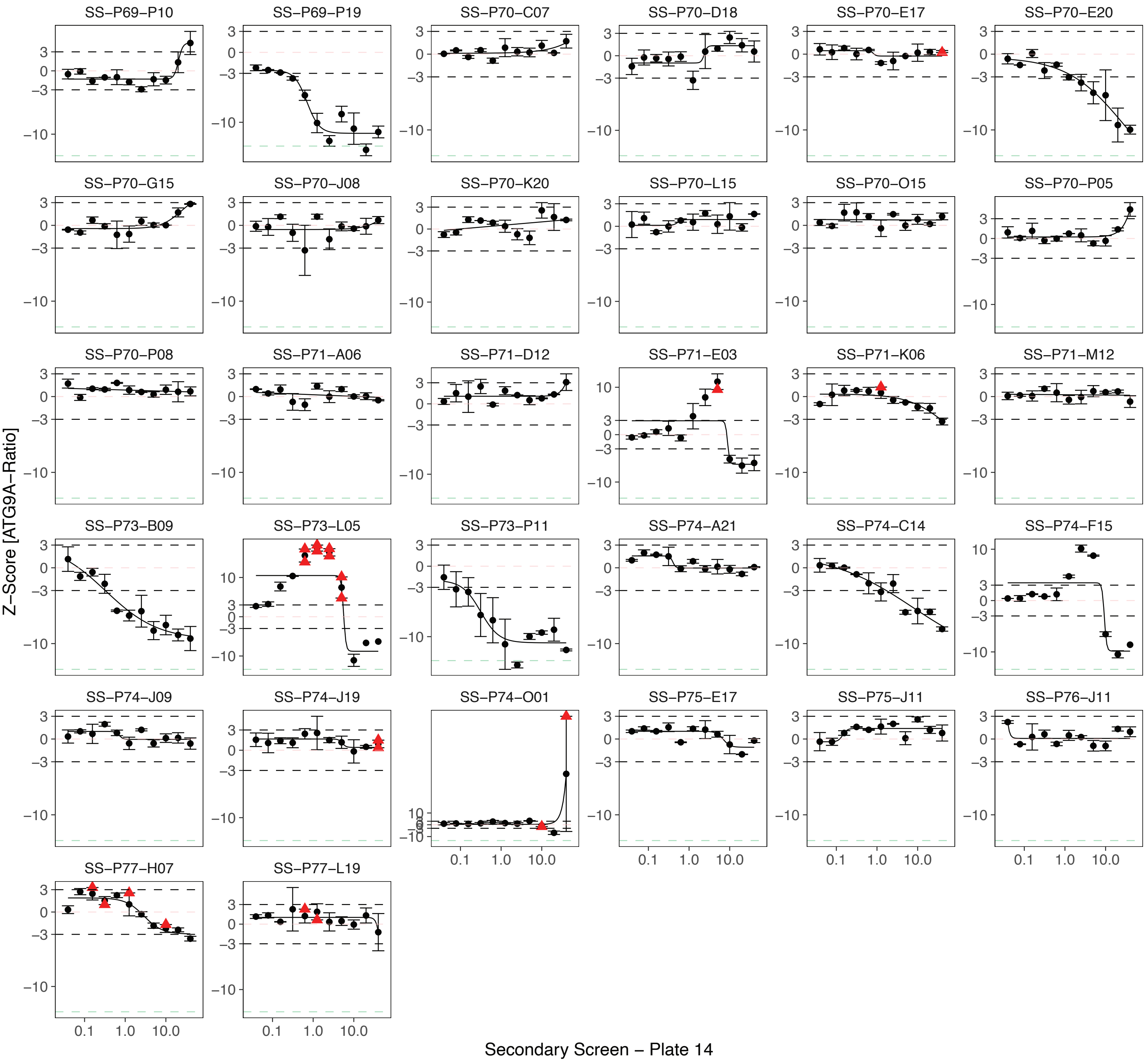
SS–P69–P10
SS–P69–P19
SS–P70–C07
SS–P70–D18
SS–P70–E17
SS–P70–E20
SS–P70–G15
SS–P70–J08
SS–P70–K20
SS–P70–L15
SS–P70–O15
SS–P70–P05
SS–P70–P08
SS–P71–A06
SS–P71–D12
SS–P71–E03
SS–P71–K06
SS–P71–M12
SS–P73–B09
SS–P73–L05
SS–P73–P11
SS–P74–A21
SS–P74–C14
SS–P74–F15
SS–P74–J09
SS–P74–J19
SS–P74–O01
SS–P75–E17
SS–P75–J11
SS–P76–J11
SS–P77–H07
SS–P77–L19
Z–Score [ATG9A–Ratio]
0.1
1.0
10.0
3
0
−3
−10
Secondary Screen – Plate 14

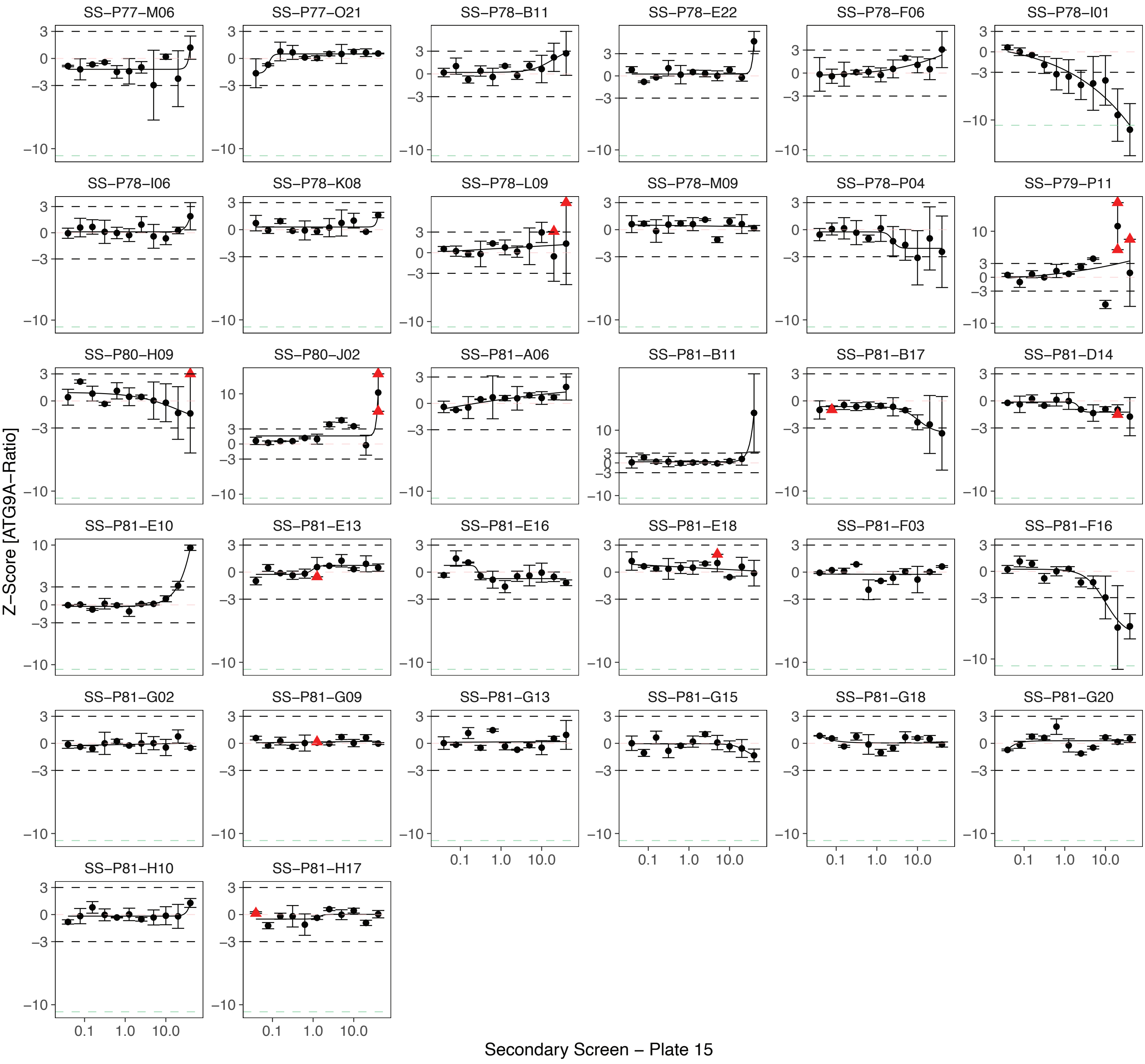
SS–P77–M06
SS–P77–O21
SS–P78–B11
SS–P78–E22
SS–P78–F06
SS–P78–I01
SS–P78–I06
SS–P78–K08
SS–P78–L09
SS–P78–M09
SS–P78–P04
SS–P79–P11
SS–P80–H09
SS–P80–J02
SS–P81–A06
SS–P81–B11
SS–P81–B17
SS–P81–D14
SS–P81–E10
SS–P81–E13
SS–P81–E16
SS–P81–E18
SS–P81–F03
SS–P81–F16
SS–P81–G02
SS–P81–G09
SS–P81–G13
SS–P81–G15
SS–P81–G18
SS–P81–G20
SS–P81–H10
SS–P81–H17
Z–Score [ATG9A–Ratio]
0.1
1.0
10.0
Secondary Screen – Plate 15

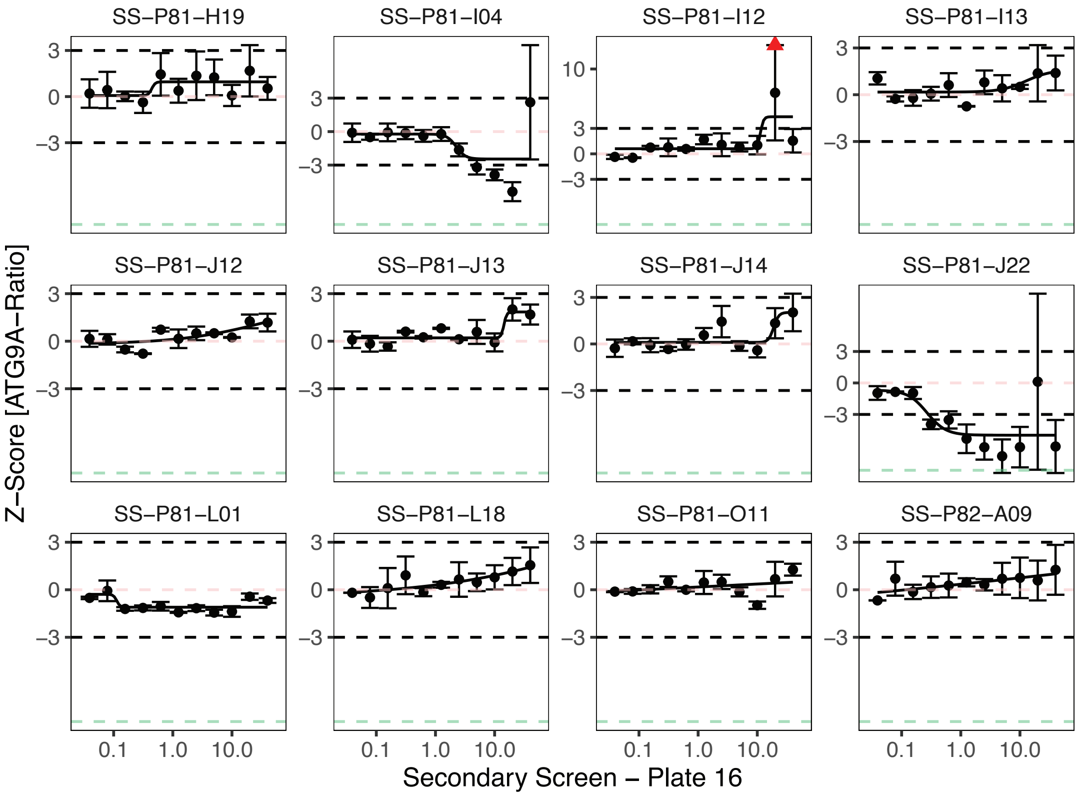

SS–P81–H19
SS–P81–I04
SS–P81–I12
SS–P81–I13
SS–P81–J12
SS–P81–J13
SS–P81–J14
SS–P81–J22
SS–P81–L01
SS–P81–L18
SS–P81–O11
SS–P82–A09
Z–Score [ATG9A–Ratio]
Secondary Screen – Plate 16

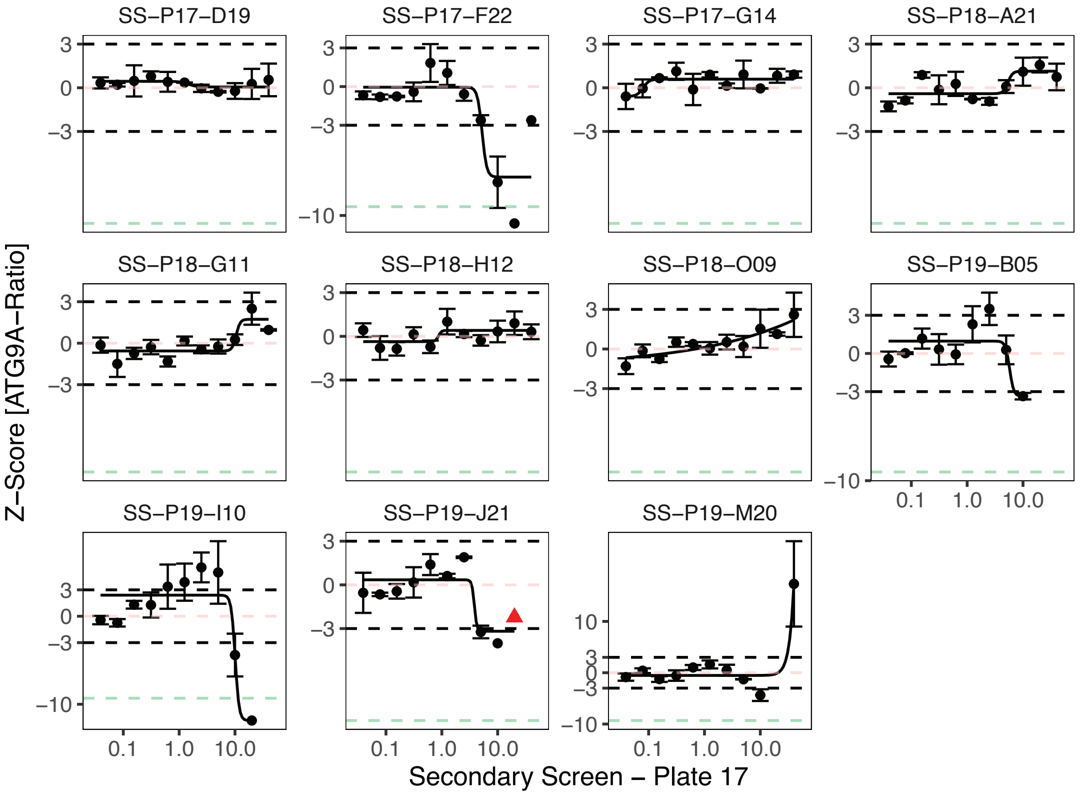

SS–P17–D19
SS–P17–F22
SS–P17–G14
SS–P18–A21
SS–P18–G11
SS–P18–H12
SS–P18–O09
SS–P19–B05
SS–P19–I10
SS–P19–J21
SS–P19–M20
Z–Score [ATG9A–Ratio]
Secondary Screen – Plate 17

Supplementary Figure 2. Summary of the counter-screen in AP-4-HSP patient-derived fibroblasts.

Overview of the counter-screen of the 503 active compounds identified in the primary screen. To assess for dose-dependent effects, compounds were screened in AP-4-HSP patient-derived fibroblasts in 384-well microplates using 11-point titrations ranging from 40 nM to 40 µM. All concentrations were screened in duplicates. Black dots and error bars represent mean ± 1 SD. Active compounds were *a priori* defined as those reducing the ATG9A ratio by at least 3 SD compared to negative controls, in more than one concentration. Toxicity was defined as a reduction of cell count of at least 2 SD compared to the negative control. Green dotted lines represent the mean of the positive controls, while red dotted lines indicate the mean of the negative controls. Red triangles indicate toxic concentrations. EC50 are indicated where possible. a) 17 compounds demonstrated a clear and reproducible dose-response relationship and raised no suspicion for autofluorescence on automated and manual review. b) 34 compounds were active but showed autofluorescence or resulted in imaging artifacts. c) Dose-response curves for all 503 compounds tested in the secondary screen.

Supplementary Figure 3.

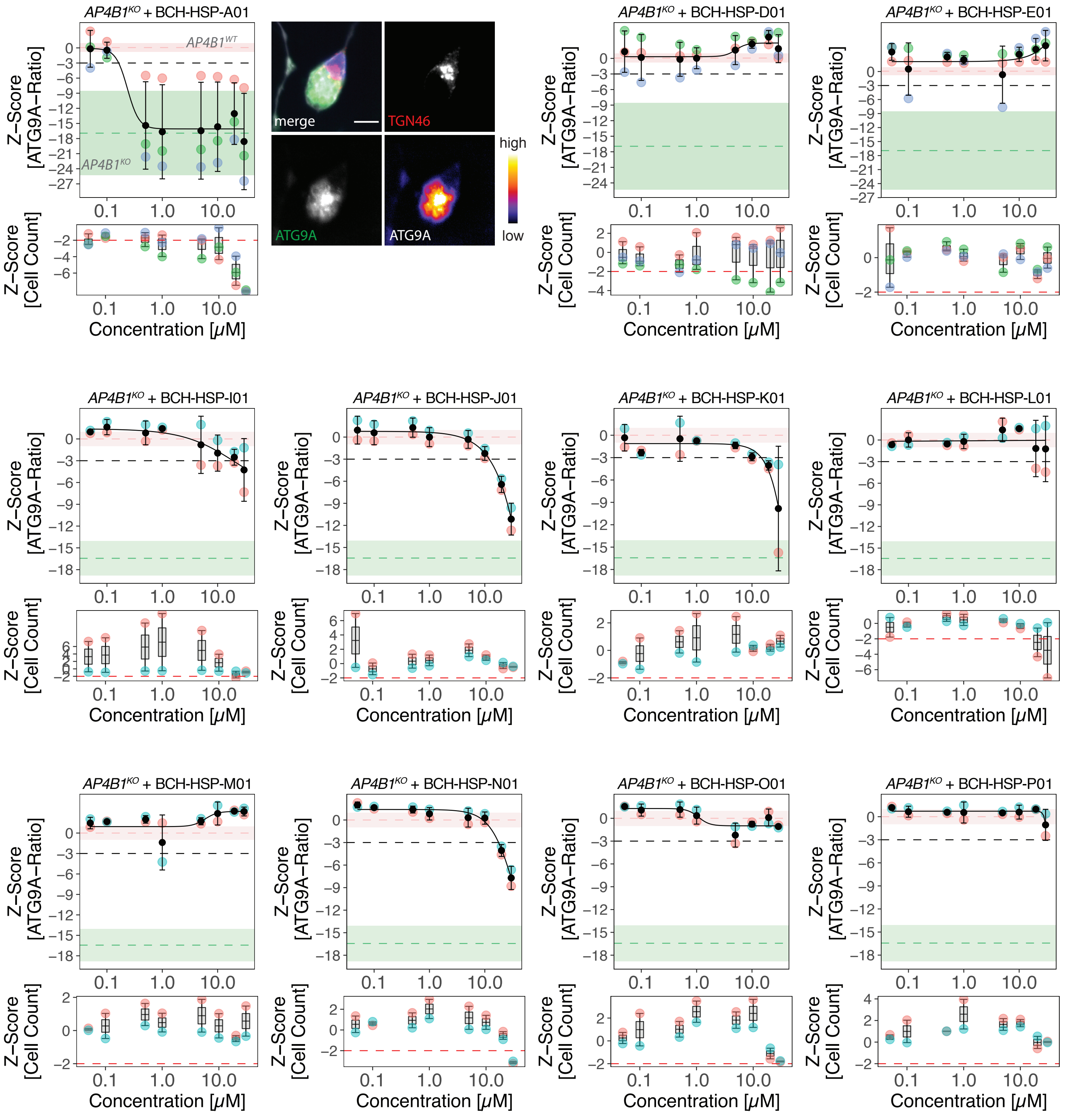

Supplementary Figure 3. Orthogonal screen identifies 11 compounds that show no activity in *AP4B1*^{KO} SH-SY5Y cells.

Dose-response curves for ATG9A ratios and cell counts in *AB4B1*^{KO} cells treated with different compounds. Data points represent per well means from 3 (BCH-HSP-A01, BCH-HSP-D01 and BCH-HSP-E01) or 2 (BCH-HSP-I01, BCH-HSP-J01, BCH-HSP-K01, BCH-HSP-L01, BCH-HSP-M01, BCH-HSP-N01, BCH-HSP-O01, BCH-HSP-P01) different assay plates. Black dots and error bars represent mean ± 1 SD. Dashed lines show mean Z-scores for positive (green) and negative (salmon) controls. Shaded areas represent ± 1 SD. Box plots show medians (center), upper and lower quartiles (hinges) and 1.5 x IQR (whiskers). Eleven of 16 compounds were excluded due to either lacking activity (*D-01*, *E-01*, *L-01*, *M-01*, *N-01*, *O-01*, *P-0*1), suspicion for artefacts or autofluorescence (*I-01*, *J-01*, *K-01*), or obvious changes in cellular morphology (*A-01*).

Supplementary Figure 4.

BCH-HSP-B01

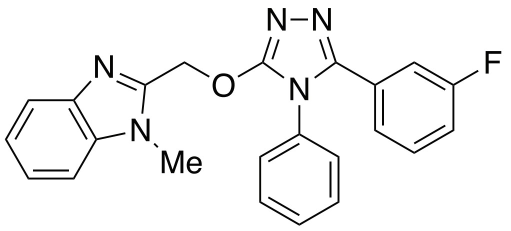

1

Chemical Formula: $C_{23}H_{18}FN_5O$
Molecular Weight: 399.43

BCH-HSP-G01

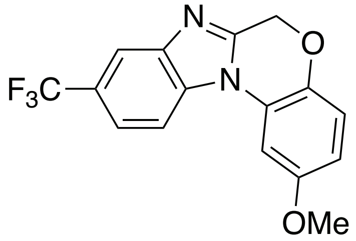

2

Chemical Formula: $C_{16}H_{11}F_3N_2O_2$
Molecular Weight: 320.27

BCH-HSP-F01

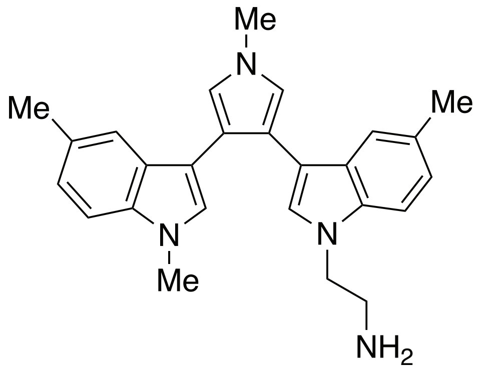

3

Chemical Formula: $C_{26}H_{28}N_4$
Molecular Weight: 396.54

BCH-HSP-H01

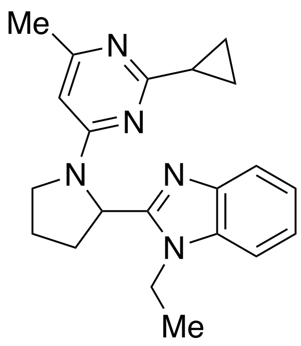

4

Chemical Formula: $C_{21}H_{25}N_5$
Molecular Weight: 347.47

BCH-HSP-C01

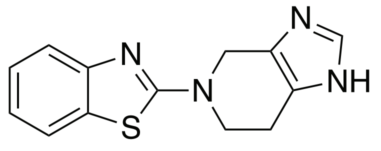

5

Chemical Formula: $C_{13}H_{12}N_4S$
Molecular Weight: 256.33

Supplementary Figure 4. Chemical structures of five active compounds.

Chemical structures of the five active compounds in *AP4B1*^{KO} SH-SY5Y cells along with their chemical formula and molecular weight.

Supplementary Figure 5.

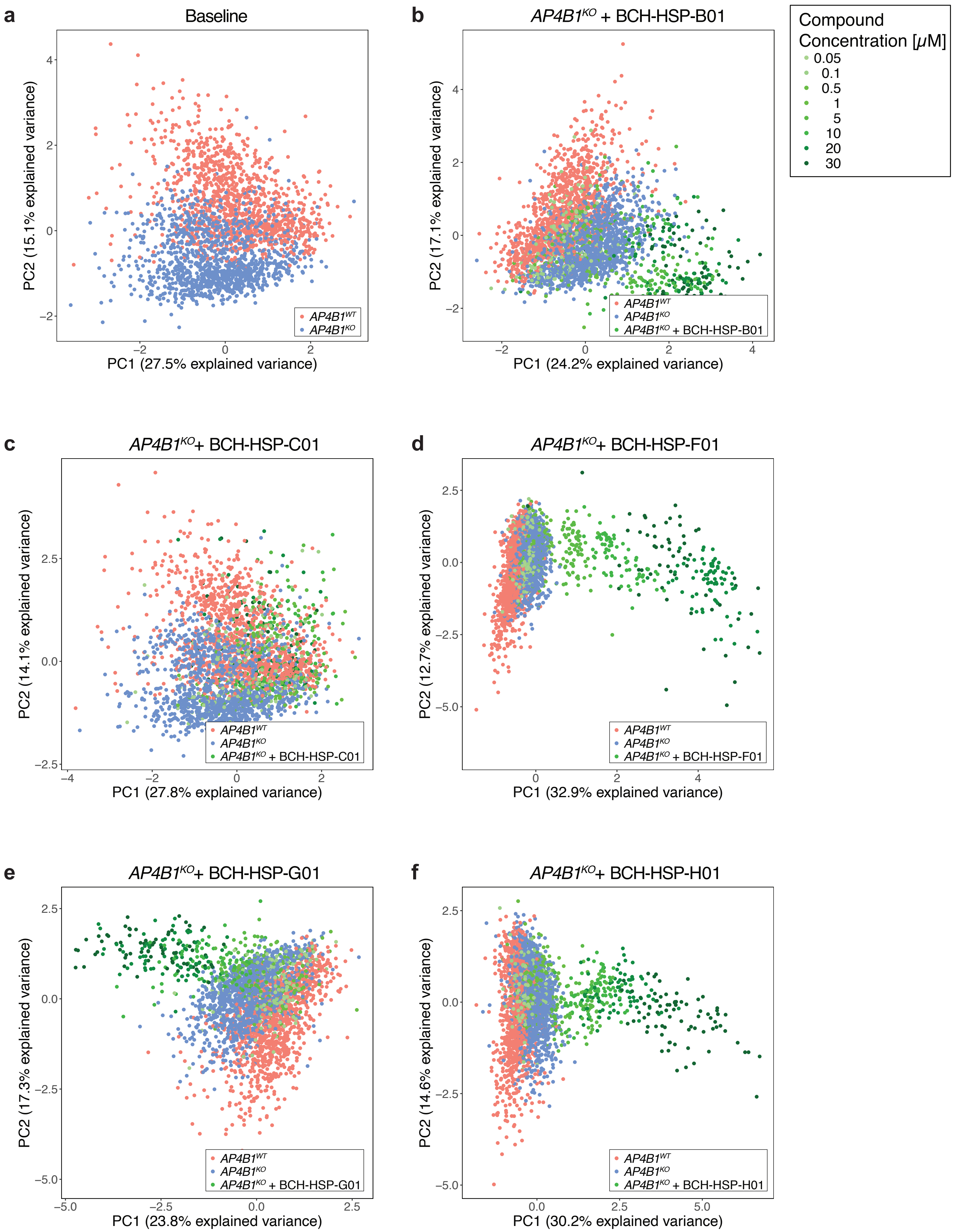

Supplementary Figure 5. Multiparametric profiling of 5 active compounds in *AP4B1^{KO}* SH-SY5Y cells.

PCA analysis of 85 extracted features of the nucleus, cytoskeleton/global cell morphology, TGN and ATG9A vesicles. (a) Baseline analysis of *AP4B1^{WT} and AP4B1^{KO}* cells. Cell lines clustered closely together and were only separated by the ATG9A signal. (b-f) Spatial clustering of the 5 active compounds in relation to the positive and negative controls. Compound concentrations are depicted by the legend. While BCH-HSP-C01 treated *AP4B1^{KO}* cells clustered closely with the controls (c), suggesting no significant off-target effects, all other compounds led to changes in overall cellular morphology in a dose-dependent manner. The most significant changes were seen for BCH-HSP-F01 (d) and BCH-HSP-H01 (f), suggesting off-target effects.

Supplementary Figure 6.

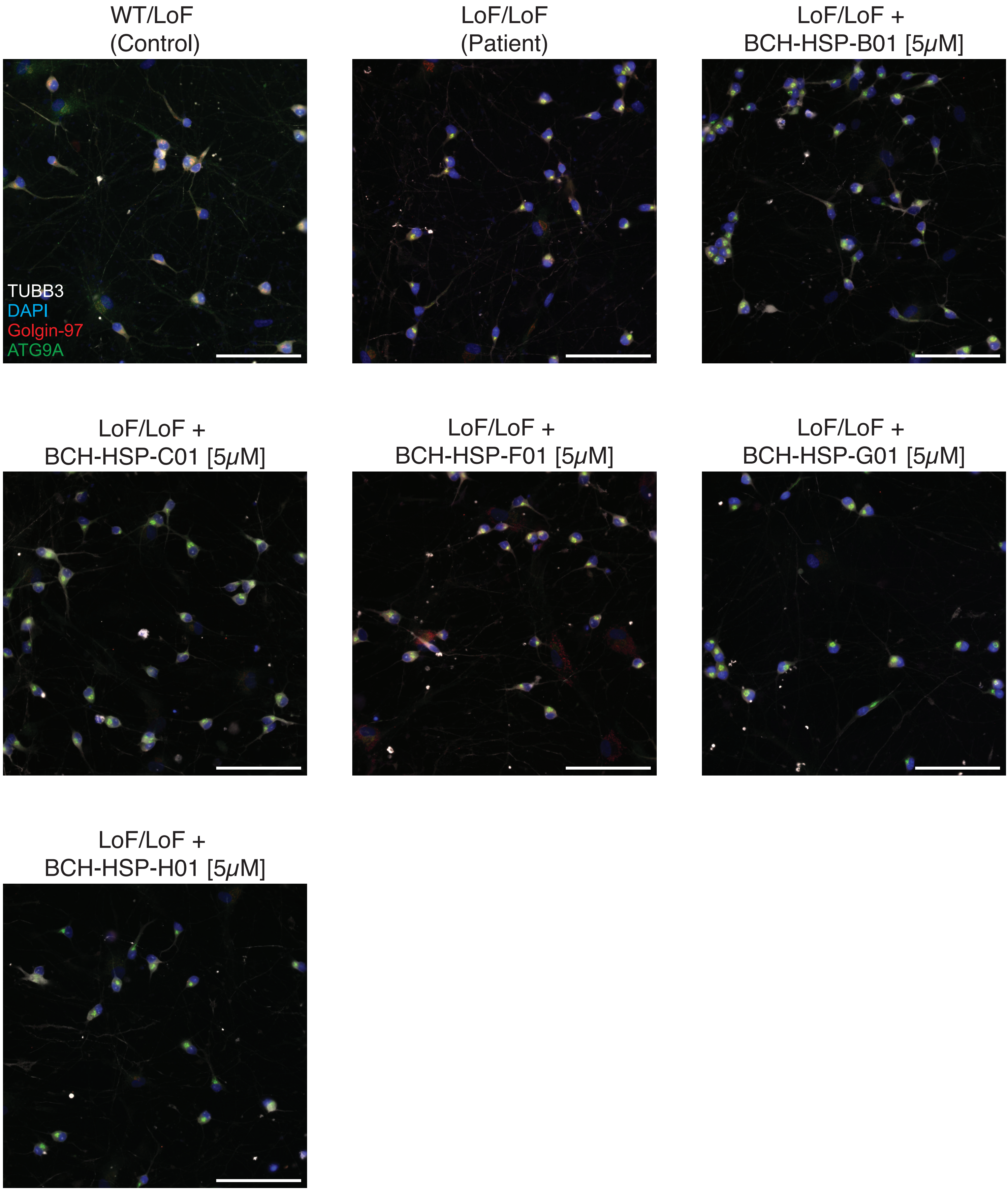

Supplementary Figure 6. Low magnification images of AP-4-HSP patient-derived hiPSC-neurons treated with 5 active compounds.

Low magnification images of the testing of 5 active compounds in hiPSC-derived cortical neurons from a patient with *AP4M1*-associated SPG50 compared to a heterozygous control (same-sex parent). The merge shows beta-3 tubulin (grey), DAPI (blue), the Golgi (red) and ATG9A (green). Scale bar: 100 μm.

Supplementary Figure 7.

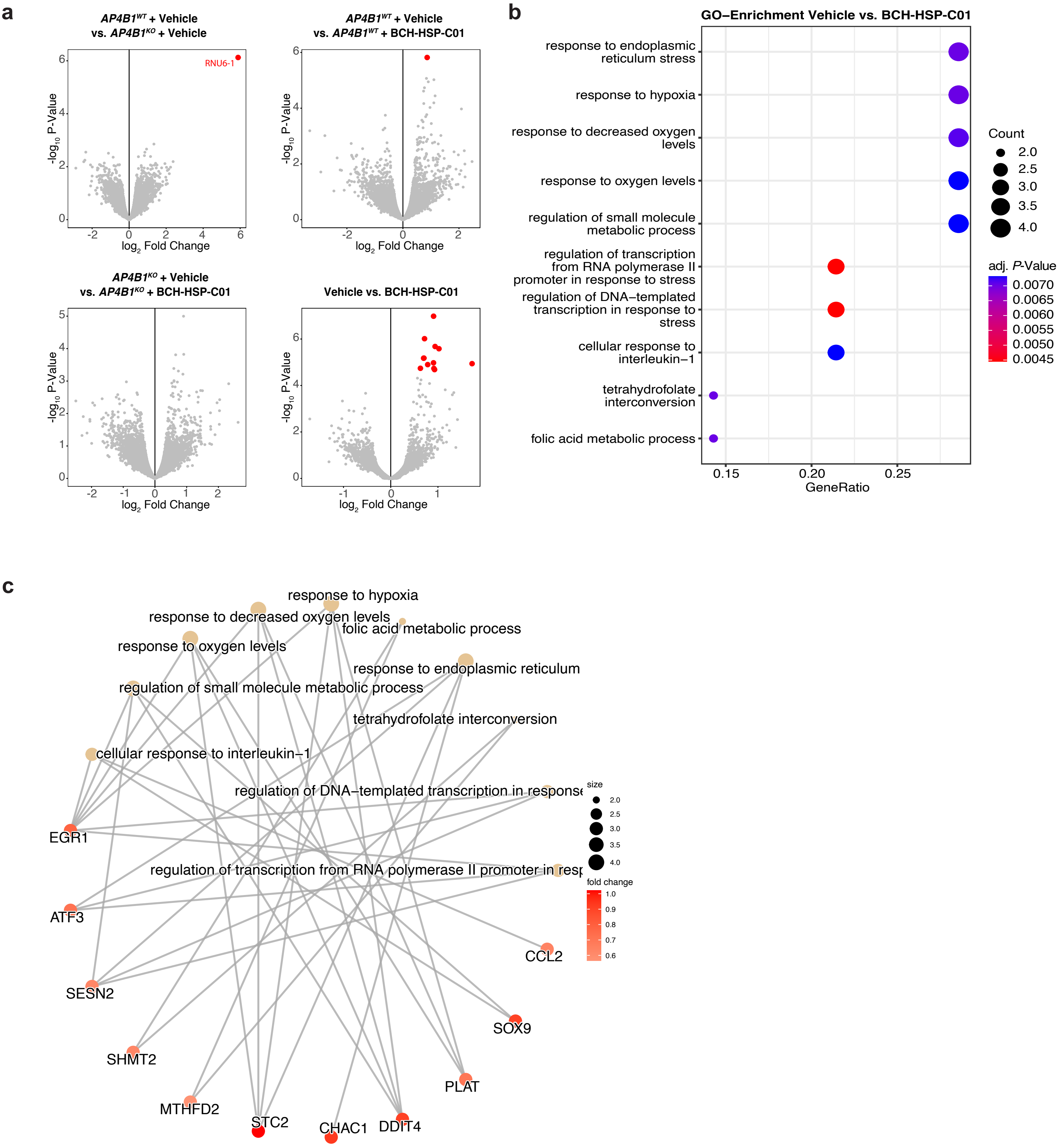

Supplementary Figure 7. Bulk RNA sequencing in *AP4B1^{KO}* SH-SY5Y cells treated with BCH-HSP-C01 shows a small number of differentially expressed genes, mainly involved in ER stress rese.

(a) Volcano plots depicting the results of bulk RNA Sequencing in different experimental conditions in SH-SY5Y cells (*AP4B1^{WT}* vs. *AP4B1^{KO}* treated with vehicle, *AP4B1^{WT}* treated with vehicle vs. *AP4B1^{WT}* treated with BCH-HSP-C01, *AP4B1^{KO}* treated with vehicle vs. *AP4B1^{KO}* treated with BCH-HSP-C01, *AP4B1^{WT}* and *AP4B1^{KO}* cells pooled in two groups, vehicle vs. BCH-HSP-C01). Differential expression analysis was done following the TREAT approach developed by McCarthy and Smyth (2009). Red colored dots represent differentially expressed genes with a $\log_2$ fold change > 0.3 and a FDR < 0.05. (b) Gene ontology analysis shows enriched pathways of the pooled analysis. Pathways were considered differentially expressed with a FDR < 0.05. (c) Gene-Concept Network showing differentially expressed genes and their pathway membership.

Supplementary Figure 8.

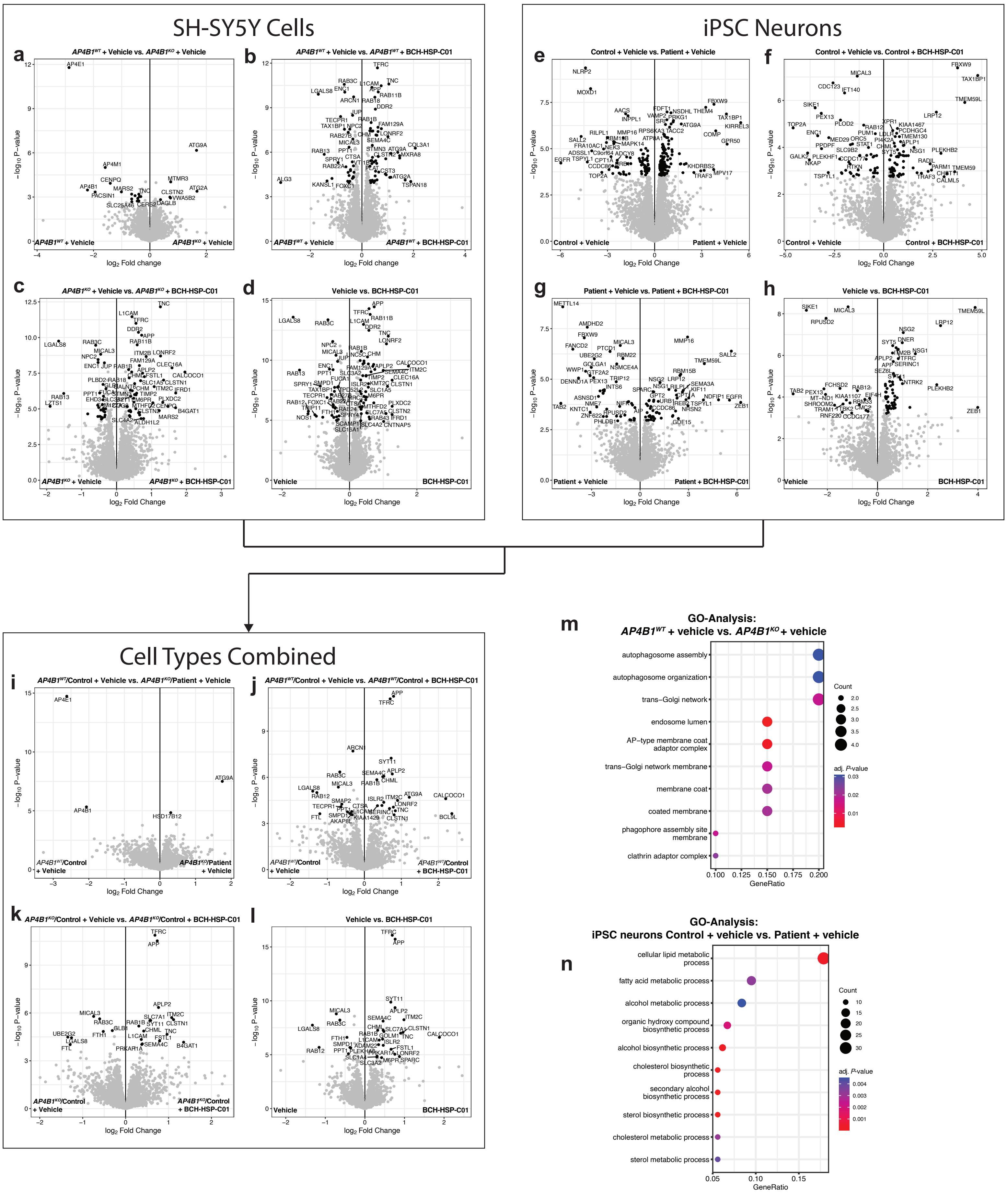

Supplementary Figure 8. Unbiased quantitative proteomics in *AP4B1^{KO}* SH-SY5Y cells and AP-4-HSP patient-derived hiPSC-neurons treated with BCH-HSP-C01.

(a-d) SH-SY5Y cells: 8141 unique proteins were analyzed. Volcano plots summarize differential protein enrichment for different experimental conditions: (a) *AP4B1^{WT}* vs. *AP4B1^{KO}* treated with vehicle, (b) *AP4B1^{WT}* treated with vehicle vs. *AP4B1^{WT}* treated with BCH-HSP-C01, (c) *AP4B1^{KO}* treated with vehicle vs. *AP4B1^{KO}* treated with BCH-HSP-C01, (d) *AP4B1^{WT}* and *AP4B1^{KO}* cells pooled treated with vehicle vs. BCH-HSP-C01. Differentially enriched proteins are depicted in black. (e-h) hiPSC-derived neurons: 7386 unique proteins were analyzed. Volcano plots summarize differential protein enrichment for different experimental conditions: (e) controls vs. patient-derived neurons treated with vehicle, (f) controls treated with vehicle vs. controls treated with BCH-HSP-C01, (g) patient-derived neurons treated with vehicle vs. patient-derived neurons treated with BCH-HSP-C01, (h) controls and patient-derived neurons pooled in two groups, treated with vehicle vs. BCH-HSP-C01. Differentially enriched proteins are depicted in black. (i-l) Integrated analysis of SH-SY5Y cells and hiPSC-derived neurons: 5357 unique proteins were analyzed. Volcano plots summarize differential protein enrichment for different experimental conditions: (i) controls vs. AP-4-deficient cells treated with vehicle, (j) controls treated with vehicle vs. controls treated with BCH-HSP-C01, (k) AP-4-deficient cells treated with vehicle vs. AP-4-deficient cells treated with BCH-HSP-C01, (l) controls and AP-4-deficient cells pooled into two groups, vehicle vs. BCH-HSP-C01. Differentially enriched proteins are depicted in black. In all datasets statistical testing for differential protein enrichment was done using protein-wise linear models and empirical Bayes statistics. Proteins were considered as differentially enriched with a FDR < 0.05 and a $\log_2$ fold change > 0.3. (m & n) Gene ontology analysis of *AP4B1^{WT}* vs. *AP4B1^{KO}* SH-SY5Y cells (m) as well as control vs. patient hiPSC-derived neurons (n) treated with vehicle shows enrichment of various cellular processes. Pathways were considered differentially enriched with a FDR < 0.05.

Supplementary Figure 9.

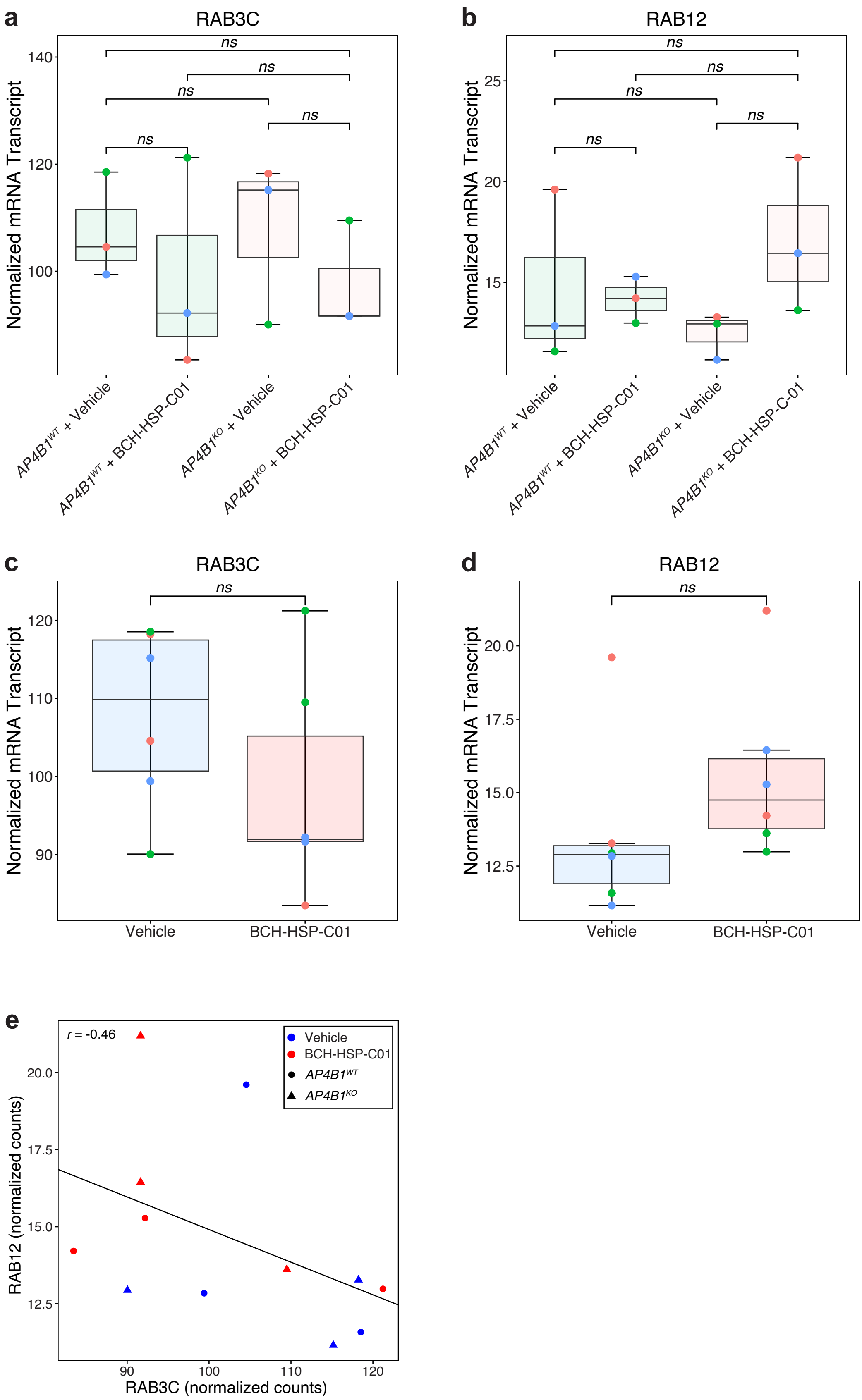

Supplementary Figure 9. mRNA transcript expression and correlation analysis of *RAB3C* and *RAB12*.

Normalized mRNA transcript counts for RAB3C (a, c) and RAB12 (b, d) across different experimental conditions in SH-SY5Y cells (*AP4B1*^{WT} treated with vehicle, *AP4B1*^{WT} treated with BCH-HSP-C01, *AP4B1*^{KO} treated with vehicle, *AP4B1*^{KO} treated with BCH-HSP-C01) (a, b), as well as *AP4B1*^{WT} and *AP4B1*^{KO} cells pooled treated with vehicle vs. BCH-HSP-C01 (c, d). No statistically significant differences were detected. Statistical testing was done using pairwise T-tests. *P*-values have been adjusted for multiple testing using the Benjamini-Hochberg procedure. (e) Correlation analysis of *RAB3C* and *RAB12* gene expression in *AP4B1*^{WT} (n = 6 samples) and *AP4B1*^{KO} (n = 6 samples) SH-SY5Y cells shows a moderate inverse correlation measured by the Pearson correlation coefficient (*r*).

Supplementary Figure 10.

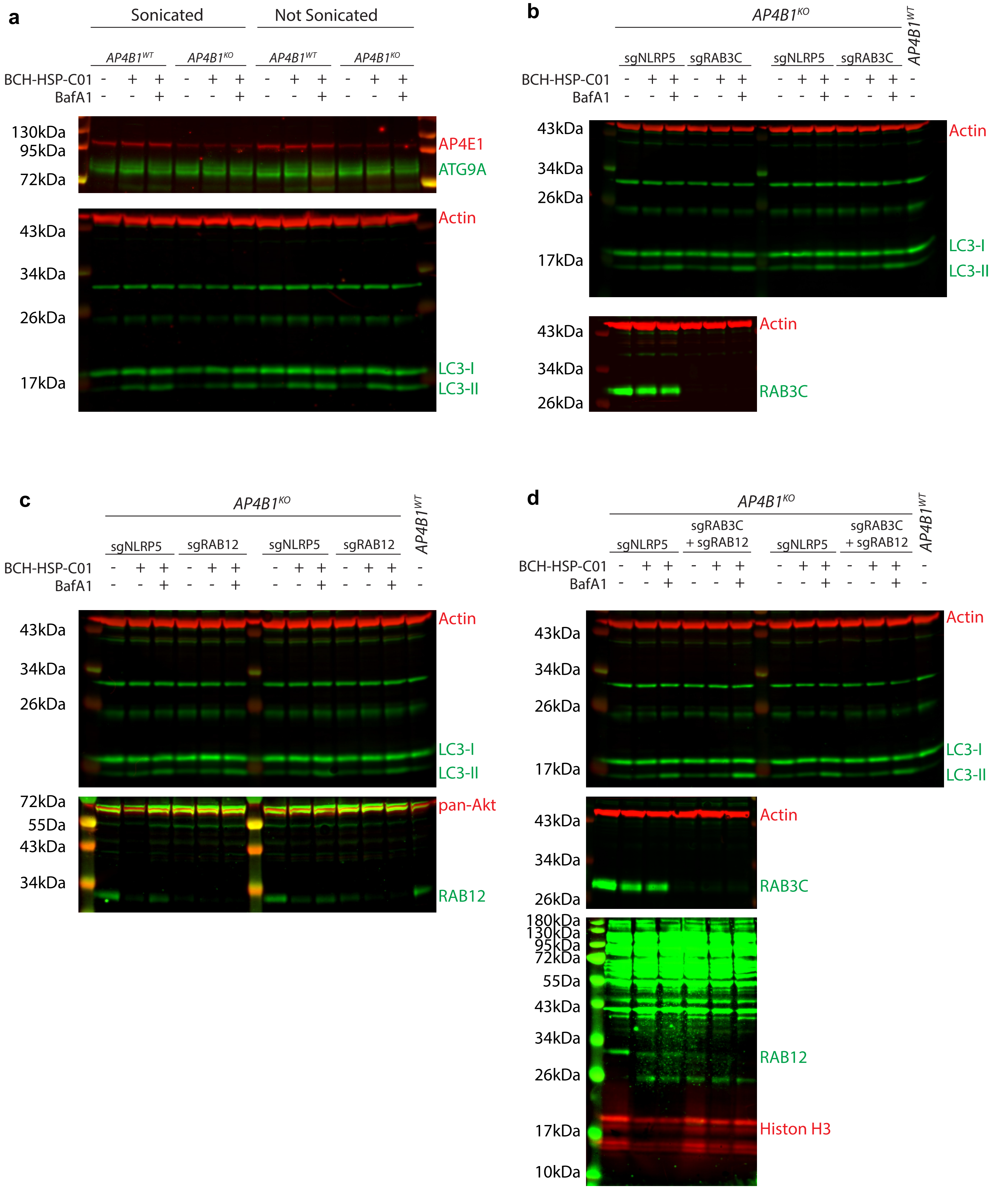

Supplementary Figure 10. Original Western blots.

(a) Uncropped, original blots corresponding to Fig. 8c. (b) Uncropped, original blot corresponding to Figure 8j. (c) Uncropped, original blot corresponding to Fig. 8k. (d) Uncropped, original blot corresponding to Fig. 8l.
