# Supplementary material for: High-content screening identifies a small molecule that restores AP-4-dependent protein trafficking in neuronal models of AP-4-associated hereditary spastic paraplegia
Source: Nat Commun. 2024 Jan 17;15:584. doi: 10.1038/s41467-023-44264-1 (PMC10794252; doi:10.1038/s41467-023-44264-1)
Supplement: Supplementary file 3 — Reporting Summary [file 41467_2023_44264_MOESM3_ESM.pdf]

nature portfolio

Corresponding author(s): Ebrahimi-Fakhari

Last updated by author(s): 12/03/2023

Reporting Summary

Nature Portfolio wishes to improve the reproducibility of the work that we publish. This form provides structure for consistency and transparency in reporting. For further information on Nature Portfolio policies, see our Editorial Policies and the Editorial Policy Checklist.

Statistics

For all statistical analyses, confirm that the following items are present in the figure legend, table legend, main text, or Methods section.

n/a	Confirmed	
☐	☒	The exact sample size (*n*) for each experimental group/condition, given as a discrete number and unit of measurement
☐	☒	A statement on whether measurements were taken from distinct samples or whether the same sample was measured repeatedly
☐	☒	The statistical test(s) used AND whether they are one- or two-sided<br>*Only common tests should be described solely by name; describe more complex techniques in the Methods section.*
☐	☒	A description of all covariates tested
☐	☒	A description of any assumptions or corrections, such as tests of normality and adjustment for multiple comparisons
☐	☒	A full description of the statistical parameters including central tendency (e.g. means) or other basic estimates (e.g. regression coefficient) AND variation (e.g. standard deviation) or associated estimates of uncertainty (e.g. confidence intervals)
☐	☒	For null hypothesis testing, the test statistic (e.g. *F*, *t*, *r*) with confidence intervals, effect sizes, degrees of freedom and *P* value noted<br>*Give P values as exact values whenever suitable.*
☒	☐	For Bayesian analysis, information on the choice of priors and Markov chain Monte Carlo settings
☐	☒	For hierarchical and complex designs, identification of the appropriate level for tests and full reporting of outcomes
☐	☒	Estimates of effect sizes (e.g. Cohen's *d*, Pearson's *r*), indicating how they were calculated

Our web collection on statistics for biologists contains articles on many of the points above.

Software and code

Policy information about availability of computer code

Data collection	Raw microscopy images were acquired using commercially available software: MetaXpress (Molecular Devices). Western blots were developed using: Odyssey infrared imaging system and Empiria Studio Software (LI-COR Biosciences). Statistical analysis of continuous variables was performed with R version 4.2.1 (2022-06-23) and Rstudio (version 2022.07.1; Rstudio, Inc.).
Data analysis	High content imaging analysis was performed using a customized image analysis pipeline in MetaXpress (Molecular Devices). Western blot quantification was done using the Odyssey infrared imaging system and Empiria Studio Software (LI-COR Biosciences). multiparametric morphological profiling strategy was performed in R version 4.2.1 (2022-06-23) and Rstudio (version 2022.07.1; Rstudio, Inc.). Differential expression analysis of RNA sequencing data was done using the TREAT approach developed by McCarthy and Smyth, implemented in the edgeR package in R. Weighted gene co-expression network analysis (WGCNA) using the limma package in R. The biological information contained in modules of interest was summarized with gene ontology (GO) enrichment analysis using clusterProfiler. Statistical analysis of continuous variables was performed with R version 4.2.1 (2022-06-23) and Rstudio (version 2022.07.1; Rstudio, Inc.)

For manuscripts utilizing custom algorithms or software that are central to the research but not yet described in published literature, software must be made available to editors and reviewers. We strongly encourage code deposition in a community repository (e.g. GitHub). See the Nature Portfolio guidelines for submitting code & software for further information.

Data

Policy information about availability of data

All manuscripts must include a data availability statement. This statement should provide the following information, where applicable:

- Accession codes, unique identifiers, or web links for publicly available datasets
- A description of any restrictions on data availability
- For clinical datasets or third party data, please ensure that the statement adheres to our policy

RNA sequencing data will be made publicly available through the National Center for Biotechnology Information's Sequence Read Archive (SRA) [accession number: PRJNA985061]. Mass spectrometry proteomics data will be deposited to the ProteomeXchange Consortium [accession number: PXD042950] via the PRIDE partner repository. Data tables with source data are provided in the supplementary material. Source images are available from the author upon reasonable request. All fibroblast and hiPSC lines generated in this study are available with a material transfer agreement.

Research involving human participants, their data, or biological material

Policy information about studies with human participants or human data. See also policy information about sex, gender (identity/presentation), and sexual orientation and race, ethnicity and racism.

Reporting on sex and gender	Patient derived fibroblasts and induced pluripotent stem cells have been reported previously, including the sex of the individuals (PMID: 31915823; PMID: 34087981; PMID: 31525725). Fibroblast lines were derived from two male probands. iPSC lines were derived from four male probands.
Reporting on race, ethnicity, or other socially relevant groupings	Does not apply.
Population characteristics	Does not apply.
Recruitment	Does not apply.
Ethics oversight	Boston Children's Hospital (IRB-P00033016 and IRB-P00016119)

Note that full information on the approval of the study protocol must also be provided in the manuscript.

Field-specific reporting

Please select the one below that is the best fit for your research. If you are not sure, read the appropriate sections before making your selection.

☒ Life sciences ☐ Behavioural & social sciences ☐ Ecological, evolutionary & environmental sciences

For a reference copy of the document with all sections, see nature.com/documents/nr-reporting-summary-flat.pdf

Life sciences study design

All studies must disclose on these points even when the disclosure is negative.

Sample size	No statistical method was used to predetermine sample size. The methodology for unbiased screens (primary and secondary screens) is described in detail in the methods section. For experiments in iPSC-derived neurons, sample size calculations were informed by our previously published high-throughput analyses of ATG9A localization (PMID: 31915823).
Data exclusions	Generally, quantitative image data underwent threshold filtering prior to analysis, as described in detail in the methods, with the aim to avoid unreliable or partial measurements near the detection limit of the method. Importantly, no data were excluded from the analyses as outliers, unless stated in the figure legend.
Replication	Figure 1. The primary screen was conducted in one biological replicate.<br>Figure 2. All dose-response curve experiments were performed in biological duplicates.<br>Figure 3. Dose-response curves were from four biological replicates.<br>Figure 5. All data points represent per well means of 3-4 independent differentiations (biological replicates).<br>Figure 8. Each experimental condition was tested in 18-28 wells from 3-5 independent plates (biological replicates).<br><br>Please refer to the detailed figure legends for additional details about number of conditions, data points, and replicates. This information is not included here for space reasons.
Randomization	This is not relevant to the study, as we were only comparing genetically different cells. No treatment of subjects was performed.
Blinding	The investigators were not blinded to allocation during experiments and outcome assessment. However, all image analyses were automated to avoid investigator bias. Additionally, objective imaging was achieved by focusing the microscope on an invariant marker protein first, without seeing the fluorescence channel of the protein of experimental relevance. Images were then collected for both channels in the same field of view. Thus, although the experimenter was not blinded to sample ID, he or she was effectively blinded to the critical data during

collection. This is described in the methods.

Reporting for specific materials, systems and methods

We require information from authors about some types of materials, experimental systems and methods used in many studies. Here, indicate whether each material, system or method listed is relevant to your study. If you are not sure if a list item applies to your research, read the appropriate section before selecting a response.

Materials & experimental systems

n/a	Involved in the study	
☐	☒	Antibodies
☐	☒	Eukaryotic cell lines
☒	☐	Palaeontology and archaeology
☒	☐	Animals and other organisms
☒	☐	Clinical data
☒	☐	Dual use research of concern
☒	☐	Plants

Methods

n/a	Involved in the study	
☒	☐	ChIP-seq
☒	☐	Flow cytometry
☒	☐	MRI-based neuroimaging

Antibodies

Antibodies used

The following primary antibodies were used: Anti-AP4E1 at 1:500 (BD Bioscience, Cat# 612019), anti-ATG9A at 1:500-1000 (Abcam, Cat# ab108338), anti-DAGLB at 1:500 (Abcam, Cat# 191159), anti-TGN46 at 1:800 (Bio-Rad, Cat# AHP500G), anti-Golgin 97 1:500 (Abcam, Cat# 169287), anti-beta-Tubulin III 1:1000 (Synaptic Systems, Cat# 302304 and Sigma, Cat# T8660), anti-beta-Actin 1:10,000 (Sigma, Cat# A1978-100UL), anti-SMI 312 (Biolegend, Cat # 837904), anti-pan-AKT (Cell Signaling Technology, Cat# 4691), anti-Histon H3 (Cell Signaling Technology, Cat # 9715), anti-RAB12 (Santa Cruz, Cat# sc-515613), anti-RAB3C (Santa Cruz, Cat# 107 203), anti-LC3B 1:1000 (Novus, Cat#100-2220). Fluorescently labelled secondary antibodies for immunocytochemistry were used at 1:2000 (Thermo Fisher Scientific, Cat# A11005, A-11008, A-11016, A-11073, A-21235, A-21245), for western blotting at 1:5000 (LI-COR Biosciences, Cat# 926-68022, 926-68023, 926-32212, 926-32213).

Validation

All antibodies used are commercially available. Please refer to the manufacturer's product page for validation.

Eukaryotic cell lines

Policy information about cell lines and Sex and Gender in Research

Cell line source(s)

Fibroblast lines were established from routine skin punch biopsies in both patients and their respective sex-matched heterozygous parents. Patient derived fibroblasts and induced pluripotent stem cells have been reported previously, including the sex of the individuals (PMID: 31915823; PMID: 34087981; PMID: 31525725). SH-SY5Y cells were reported previously (PMID: 35217685).

Authentication

Authentication was performed for iPSC clones in PMID: 31915823; PMID: 34087981; PMID: 31525725).

Mycoplasma contamination

All cell lines were routinely tested for mycoplasma contamination. This is also described in PMID: 31915823; PMID: 34087981; PMID: 31525725).

Commonly misidentified lines
(See ICLAC register)

No commonly misidentified cell lines were used.

Plants

Seed stocks

Report on the source of all seed stocks or other plant material used. If applicable, state the seed stock centre and catalogue number. If plant specimens were collected from the field, describe the collection location, date and sampling procedures.

Novel plant genotypes

Describe the methods by which all novel plant genotypes were produced. This includes those generated by transgenic approaches, gene editing, chemical/radiation-based mutagenesis and hybridization. For transgenic lines, describe the transformation method, the number of independent lines analyzed and the generation upon which experiments were performed. For gene-edited lines, describe the editor used, the endogenous sequence targeted for editing, the targeting guide RNA sequence (if applicable) and how the editor was applied.

Authentication

Describe any authentication procedures for each seed stock used or novel genotype generated. Describe any experiments used to assess the effect of a mutation and, where applicable, how potential secondary effects (e.g. second site T-DNA insertions, mosiacism, off-target gene editing) were examined.
