# Supplementary material for: High-content screening identifies a small molecule that restores AP-4-dependent protein trafficking in neuronal models of AP-4-associated hereditary spastic paraplegia
Source: Nat Commun. 2024 Jan 17;15:584. doi: 10.1038/s41467-023-44264-1 (PMC10794252; doi:10.1038/s41467-023-44264-1)
Supplement: Supplementary file 4 — Source Data [file 41467_2023_44264_MOESM4_ESM.zip › Description.docx]

**Description of Source Data files**

Complete datasets for the primary screen in patient-derived fibroblasts are deposited in Source Data files 1-3.

Complete datasets for the counter screen in patient-derived fibroblasts are deposited in Source Data files 4-5.

Complete datasets for orthogonal screens in *AP4B1^KO^* SH-SY5Y cells are deposited in Source Data file 6.

Complete datasets for orthogonal screens in hiPSC-neurons from AP-4-HSP patients are deposited in Source Data file 7.

Complete datasets for gene expression analysis in *AP4B1^KO^* SH-SY5Y cells are deposited in Source Data files 8-9.

Complete datasets for proteomic profiling in *AP4B1^KO^* SH-SY5Y cells and hiPSC-neurons from a patient with AP-4-HSP are deposited in Source Data file 10.

Complete datasets for knockout experiments in *AP4B1^KO^* SH-SY5Y cells are deposited in Source Data file 11.
